# Conjugations of unitary operators, II

**DOI:** 10.1007/s13324-024-00920-3

**Published:** 2024-05-10

**Authors:** Javad Mashreghi, Marek Ptak, William T. Ross

**Affiliations:** 1 Département de mathématiques et de Statistique, Université Laval, Québec, QC G1K 0A6 Canada; 2 Department of Applied Mathematics, University of Agriculture, ul. Balicka, 253c 30-198 Kraków, Poland; 3 Department of Mathematics and Computer Science, University of Richmond, Richmond, VA 23173 USA

**Keywords:** Unitary operators, Conjugations, Model spaces, Shift operators, Invariant subspaces, 47B35, 47B02, 47A05

## Abstract

For a unitary operator *U* on a separable complex Hilbert space ${\mathcal {H}}$, we describe the set ${\mathscr {C}}_{c}(U)$ of all conjugations *C* (antilinear, isometric, and involutive maps) on ${\mathcal {H}}$ for which $C U C = U$. As this set might be empty, we also show that ${\mathscr {C}}_{c}(U) \not = \varnothing$ if and only if *U* is unitarily equivalent to $U^{*}$.

## Introduction

This is the second in a series of two papers that explore conjugations of unitary operators on Hilbert spaces. The first paper [19] explored, for a given unitary operator *U* on a Hilbert space ${\mathcal {H}}$, the antilinear, isometric, and involutive maps *C* on ${\mathcal {H}}$, i.e., *conjugations*, for which $C U C = U^{*}$. An argument with the spectral theorem says there will always be a conjugation *C* with this property. Moreover, [19] contains various characterizations of the set of *all* such conjugations *C* for which $C U C = U^{*}$. These conjugations are the “symmetric conjugations” for *U*.

The purpose of this paper is to explore, for a given unitary *U* on ${\mathcal {H}}$, the set

1.1 $$\begin{aligned} {\mathscr {C}}_c(U):= \{\text{ C } \text{ is } \text{ a } \text{ conjugation } \text{ on } {\mathcal {H}}: C U C = U\}. \end{aligned}$$

These are known as the “commuting conjugations” for *U* [3, 4]. The subscript *c* in the definition of ${\mathscr {C}}_{c}(U)$ might initially seem superfluous but we will use it anyway to distinguish this set from ${\mathscr {C}}_{s}(U)$ (notice the *s* in the subscript), the “symmetric conjugations” mentioned in the previous paragraph. For an easy example of a commuting conjugation, consider the unitary operator $(U f)(\xi ) = \xi f(\xi )$, the bilateral shift on $L^2(m, \mathbb {T})$, where *m* is normalized Lebesgue measure on the unit circle $\mathbb {T}$. One can check that the map $(J f)(\xi ) = \overline{f(\overline{\xi })}$ on $L^2(m, \mathbb {T})$ defines a conjugation which satisfies $J U J = U$. Moreover (see Example 6.16), any conjugation *C* on $L^2(m, \mathbb {T})$ for which $C U C = U$ takes the form $(C f)(\xi ) = u(\xi ) (J f)(\xi )$, where $u \in L^{\infty }(m, \mathbb {T})$ is both unimodular and satisfies $u(\xi ) = u(\overline{\xi })$ a.e.  on $\mathbb {T}$. An analogous result holds when $(U {\varvec{f}})(\xi ) = \xi {\varvec{f}}(\xi )$ on the vector-valued Lebesgue space ${\mathscr {L}}^2(m, {\mathcal {H}})$, but not always on ${\mathscr {L}}^2(\mu , {\mathcal {H}})$ for a general positive measure $\mu$ on $\mathbb {T}$ (see Sect. 4 and the discussion below).

The first issue one needs to resolve is whether, for a given unitary operator *U* on ${\mathcal {H}}$, there are *any* conjugations *C* for which $C U C = U$. Indeed, using the known fact from [16] (see also Proposition 2.8 below) that any unitary operator can be written as a composition of two conjugations, one can fashion a quick argument (see Lemma 2.9) to see that if ${\mathscr {C}}_{c}(U) \not = \varnothing$, then $U \cong U^{*}$ (i.e., *U* is unitarily equivalent to its adjoint $U^{*}$). One of the main results of this paper (Corollary 5.4) is the converse.

### Theorem 1.2

For a unitary operator *U* on a complex separable Hilbert space ${\mathcal {H}}$, the following are equivalent.

- $${\mathscr {C}}_{c}(U) \not = \varnothing $$;
- $$U \cong U^{*}$$.

Notice how condition (b) in Theorem 1.2 places some restrictions on the class of unitary operators which have commuting conjugations in that, at the very least, the spectrum $\sigma (U)$ of *U* must be symmetric with respect to the real axis.

We give concrete descriptions of ${\mathscr {C}}_{c}(U)$ for many classes of unitary operators *U* such as the bilateral shift on $L^2(m)$, the related bilateral shift on $L^2(\mu )$, the bilateral shift on the vector-valued ${\mathscr {L}}^2(\mu , {\mathcal {H}})$, multiplication by an inner function on $L^2(m)$, general bilateral shifts, the Fourier transform, and the Hilbert transform. The main driver all of these results comes from the classical spectral theorem for unitary operators (multiplicity version) [5, p.  307, Ch. IX, Theorem 10.20] which says that any unitary operator *U* on ${\mathcal {H}}$ is unitarily equivalent to

1.3 $$\begin{aligned} \widetilde{{{\varvec{M}}}}:= {{\varvec{M}}}_{\xi }^{(\infty )} \oplus {{\varvec{M}}}_{\xi }^{(1)} \oplus {{\varvec{M}}}_{\xi }^{(2)} \oplus \cdots , \end{aligned}$$

on

$$\begin{aligned} {{\varvec{L}}}^2_{{\mathcal {H}}}: = {\mathscr {L}}^2(\mu _\infty ,{\mathcal {H}}_\infty )\oplus {\mathscr {L}}^2(\mu _1,{\mathcal {H}}_1)\oplus {\mathscr {L}}^2(\mu _2,{\mathcal {H}}_2)\oplus \cdots \end{aligned}$$

where for $i = \infty , 1, 2, 3, \ldots$, $\mu _i$ are finite positive Borel measures on $\mathbb {T}$,

$$\begin{aligned} {{\varvec{M}}}_{\xi }^{(i)}: {\mathscr {L}}^2(\mu _{i}, {\mathcal {H}}_{i}) \rightarrow {\mathscr {L}}^2(\mu _{i}, {\mathcal {H}}_{i}), \quad ({{\varvec{M}}}_{\xi }^{(i)} {\varvec{f}})(\xi ) = \xi {\varvec{f}}(\xi ), \end{aligned}$$

and ${\mathcal {H}}_i$ are *i*-dimensional Hilbert spaces. Let ${\mathcal {I}}: {\mathcal {H}}\rightarrow {{\varvec{L}}}^2_{{\mathcal {H}}}$ denote the isometric isomorphism that induces the unitary equivalence of *U* and $\widetilde{{{\varvec{M}}}}$. In Lemma 5.2 we show that if ${\mathscr {C}}_{c}(U) \not = \varnothing$, then $\mu _i^c\ll \mu _i$ for all $i=\infty ,1,2,\dots$. Here $\mu _i^{c}(\Omega ):= \mu _i(\Omega ^{*})$, where $\Omega ^{*}: = \{\overline{\xi }: \xi \in \Omega \}.$ Using these tools, the main description of ${\mathscr {C}}_{c}(U)$ is the following. We refer the reader to Sect. 4 for the precise definitions of the parameters ${\varvec{J}}^{\#}$ and ${{\varvec{U}}}^{\#}$.

### Theorem 1.4

Let *U* be a unitary operator and *C* be a conjugation on ${\mathcal {H}}$. With the notation above, assuming that $\mu _i^c\ll \mu _i$ for $i=\infty ,1,2,\dots$, the following are equivalent

- $$C \in {\mathscr {C}}_{c}(U)$$;
- For each $i=\infty ,1,2,\dots$, there are conjugations ${{\varvec{C}}}^{i}$ on ${\mathscr {L}}^{2}(\mu _i,{\mathcal {H}}_i)$ such that ${{\varvec{M}}}_\xi ^{(i)}$ is ${{\varvec{C}}}^{i}$–commuting and $C={{\mathcal {I}}}^{*}\Big (\bigoplus {{\varvec{C}}}^i\Big ){\mathcal {I}};$
- For each $i=\infty ,1,2,\dots$ and any conjugation $J^{(i)}$ on ${\mathcal {H}}_i$, there is a unitary operator-valued function ${{\varvec{U}}}^{(i)}\in {\mathscr {L}}^{\infty }(\mu _i,{\mathcal {B}}({\mathcal {H}}_i))$ such that $J^{(i)}{{\varvec{U}}}^{(i)}(\xi )J^{(i)}={{\varvec{U}}}^{(i)}(\xi )^{\#}$ for $\mu _i$-a.e.  $\xi \in \mathbb {T}$ and
  $$\begin{aligned} C={{\mathcal {I}}}^{*}\Big (\bigoplus {{\varvec{U}}}^{(i)}{{\varvec{J}}^{\#}}^{(i)}\Big ){\mathcal {I}}={{\mathcal {I}}}^{*}\Big (\bigoplus {{\varvec{U}}}^{(i)}\Big )\Big (\bigoplus {{\varvec{J}}^{\#}}^{(i)}\Big ){\mathcal {I}}. \end{aligned}$$

This theorem is stated and proven in Sect. 5 and the concrete characterizations of ${\mathscr {C}}_{c}(U)$, for particular classes of unitary operators, will be given in Sect. 6. Parallel to a discussion in the first paper [19] in this series, we discuss the (closed) subspaces ${\mathcal {K}}$ of ${\mathcal {H}}$ for which $C {\mathcal {K}} \subseteq {\mathcal {K}}$ for all $C \in {\mathscr {C}}_{c}(U)$ in Sect. 7.

## Basics facts about conjugations

*All Hilbert spaces* ${\mathcal {H}}$ *in this paper are separable and complex.* Let ${\mathcal {B}}({\mathcal {H}})$ denote the space of all bounded linear transformations on ${\mathcal {H}}$ and ${\mathscr {A}}\!{\mathcal {B}}({\mathcal {H}})$ denote the space of all *bounded antilinear transformations* on ${\mathcal {H}}$. By this we mean that $C \in {\mathscr {A}}\!{\mathcal {B}}({\mathcal {H}})$ when $C(\textbf{x} + \alpha \textbf{y}) = C \textbf{x} + \overline{\alpha } C \textbf{y}$ for all $\textbf{x}, \textbf{y} \in {\mathcal {H}}$ and $\alpha \in \mathbb {C}$ (*C* is antilinear) and $\sup \{\Vert C \textbf{x}\Vert : \Vert \textbf{x}\Vert = 1\}$ is finite (*C* is bounded). We say that $C \in {\mathscr {A}}\!{\mathcal {B}}({\mathcal {H}})$ is a *conjugation* if it satisfies the additional conditions that $\Vert C \textbf{x}\Vert = \Vert \textbf{x}\Vert$ for all $\textbf{x} \in {\mathcal {H}}$ (*C* is isometric) and $C^2 = I$ (*C* is involutive). By the polarization identity, a conjugation also satisfies

2.1 $$\begin{aligned} \langle C \textbf{x}, C \textbf{y}\rangle = \langle \textbf{y}, \textbf{x}\rangle \; \text{ for } \text{ all } \textbf{x}, \textbf{y} \in {\mathcal {H}}. \end{aligned}$$

Conjugations play an important role in operator theory and were initially studied in [8, 9, 11–13]. More recently, conjugations were explored in [3, 4, 6, 7, 18, 21].

### Example 2.2

Many types of conjugations were outlined in [11–13]. Below are a few basic ones that are relevant to this paper.

- The mapping $C f = {\bar{f}}$ defines a conjugation on a standard Lebesgue space $L^2(\mu , X)$. In particular, the mapping $C\textbf{x} = \overline{\textbf{x}}$ defines a conjugation on Euclidean space $\mathbb {C}^n$. Throughout this paper we will use the superscript *t* to represent the transpose of a matrix. In addition, vectors $\textbf{x}$ in $\mathbb {C}^n$ will be viewed as column vectors since, for an $n \times n$ matrix *A* of complex numbers, we will often consider linear transformations on $\mathbb {C}^n$ defined by $\textbf{x} \mapsto A \textbf{x}$.
- The mapping $(C f)(\xi ) = \overline{f(\overline{\xi })}$ defines a conjugation on $L^2(\mu , \mathbb {T})$ for any finite positive Borel measure on $\mathbb {T}$.
- On $L^2(\mathbb {R})$ one can consider the two conjugations $(C f)(t) = \overline{f(t)}$ and $(C f)(t) = \overline{f(-t)}$. These were used in [1, 2] to study symmetric operators and their connections to physics.

This next lemma enables us to transfer a conjugation on one Hilbert space to a conjugation on another. The (easy) proof is left to the reader.

### Lemma 2.3

Suppose ${\mathcal {H}}$ and ${\mathcal {K}}$ are Hilbert spaces and $V: {\mathcal {H}} \rightarrow {\mathcal {K}}$ is a unitary operator. If *C* is a conjugation on ${\mathcal {H}}$ then $VCV^{*}$ is a conjugation on ${\mathcal {K}}$.

### Example 2.4

We have already discussed the how the mapping $(C f)(\xi ) = \overline{f(\overline{\xi })}$ on $L^2(m, \mathbb {T})$ is a conjugation that commutes with the bilateral shift $(U f)(\xi ) = \xi f(\xi )$. Here are a few other examples.

- The conjugation $(C f)(x) = \overline{f(x)}$ on $L^2(\mathbb {R})$ commutes with the unitary operator $(U f)(x) = f(x - 1)$. This conjugation also commutes with the Hilbert transform.
- The conjugation $(C f)(x) = \overline{f(-x)}$ on $L^2(\mathbb {R})$ commutes with the Fourier–Plancherel transform.

Recalling the definition of ${\mathscr {C}}_{c}(U)$ from (1.1), let us make a few elementary observations. One can argue from (2.1) that

2.5 $$\begin{aligned} {\mathscr {C}}_{c}(U) = {\mathscr {C}}_{c}(U^{*}). \end{aligned}$$

Commuting conjugations are stable under unitary equivalence.

### Proposition 2.6

Suppose *U*, *V*, *W* are unitary operators on ${\mathcal {H}}$ such that $W U W^{*} = V$. Then $W {\mathscr {C}}_{c}(U) W^{*} = {\mathscr {C}}_{c}(V)$.

If *U* is unitary and *C* is a conjugation on ${\mathcal {H}}$, then $UC \in {\mathscr {A}}\!{\mathcal {B}}({\mathcal {H}})$ and is isometric. This next result has a straightforward proof and determines when *UC* is involutive and hence a conjugation.

### Lemma 2.7

Let *U* be a unitary operator and *C* be a conjugation on ${\mathcal {H}}$. Then *UC* is a conjugation if and only if $C U C = U^{*}$.

We recall the following result from [16] (see also Proposition 2.5 from [19]) which shows that any unitary operator can be built from conjugations.

### Proposition 2.8

Let *U* be a unitary operator on ${\mathcal {H}}$. Then there are conjugations $J_1$ and $J_2$ on ${\mathcal {H}}$ such that $U=J_1J_2.$ Moreover, $J_1 U J_1 = U^{*}$ and $J_2 U J_2 = U^{*}$.

In the introduction we showed that although every unitary operator *U* satisfies $C U C = U^{*}$ with respect to some conjugation *C*, it is possible for ${\mathscr {C}}_{c}(U)$ (the commuting conjugations for *U*) to be the empty set. Below we begin to determine when this happens (and bring this discussion to fruition in Corollary 5.4).

### Lemma 2.9

If *U* is a unitary operator on ${\mathcal {H}}$ and ${\mathscr {C}}_{c}(U) \not = \varnothing$, then $U \cong U^{*}$.

### Proof

Let $J_1$ be as in Proposition 2.8, $C \in {\mathscr {C}}_{c}(U)$, and define $V=J_1C$. Clearly *V* is unitary (since it is linear, isometric, and onto) and $VU =J_1CU=J_1UC=U^{*}J_1C=U^*V$. Thus, $U \cong U^{*}$. $\square$

## Conjugations and spectral measures

A version of the spectral theorem for unitary operators [5, Ch. IX, Thm. 2.2] (see also [17]) says that if *U* is a unitary operator on ${\mathcal {H}}$, then there is a unique spectral measure $E(\cdot )$ on $\mathbb {T}$ such that

3.1 $$\begin{aligned} U = \int \xi d E(\xi ). \end{aligned}$$

Moreover, for any spectral measure $E(\cdot )$ on $\mathbb {T}$, there is a unique unitary operator *U* associated with $E(\cdot )$ via (3.1).

For a spectral measure $E(\cdot )$ and $\textbf{x}, \textbf{y} \in {\mathcal {H}}$, the function

$$\begin{aligned} \mu _{\textbf{x}, \textbf{y}}(\cdot ):= \langle E(\cdot ) \textbf{x}, \textbf{y}\rangle \end{aligned}$$

defines a finite complex Borel measure on $\mathbb {T}$ and for each $\textbf{x} \in {\mathcal {H}}$,

$$\begin{aligned} \mu _{\textbf{x}}:=\mu _{\textbf{x},\textbf{x}} \end{aligned}$$

defines a finite positive Borel measure on $\mathbb {T}$, called an *elementary measure*.

For a complex Borel measure $\mu$ on $\mathbb {T}$, define a new Borel measure $\mu ^{c}$ on the Borel subsets $\Omega$ of $\mathbb {T}$ by

3.2 $$\begin{aligned} \mu ^{c}(\Omega ):= \mu (\Omega ^{*}), \; \; \text{ where } \; \; \Omega ^{*}: = \{\overline{\xi }: \xi \in \Omega \} \end{aligned}$$

It is clear that $(\mu ^{c})^{c} = \mu$. For a spectral measure $E(\cdot )$ on $\mathbb {T}$, we have the family of measures $\{\mu _{\textbf{x}, \textbf{y}}^{c}: \textbf{x}, \textbf{y} \in {\mathcal {H}}\}$ defined via (3.2).

For the rest of this paper, we use $M_{+}(\mathbb {T})$ to denote the set of all finite positive Borel measures on $\mathbb {T}$.

### Proposition 3.3

Suppose $\mu \in M_{+}(\mathbb {T})$ and $\mu ^c \ll \mu$. Then the following hold.

- $$\mu \ll \mu ^{c}$$;
- The Radon–Nikodym derivatives satisfy
  $$\begin{aligned} \frac{d\mu ^c}{d\mu }(\xi ) \cdot \frac{d\mu ^c}{d\mu }(\bar{\xi })=1 \; \; \hbox {for } \mu \hbox {-a.e.}~ \xi \in \mathbb {T}. \end{aligned}$$

### Proof

Let $h = d \mu ^{c}/d \mu$. Observe that $\mu =(\mu ^c)^c\ll \mu ^c$ and

$$\begin{aligned} d \mu (\xi )= d \mu ^c(\bar{\xi }) = h(\bar{\xi })d \mu (\bar{\xi }) = h(\bar{\xi })d \mu ^{c}(\xi ) = h(\bar{\xi }) h(\xi )d \mu (\xi ) \; \text{ for } \mu \text{-a.e. } \xi \in \mathbb {T}\text{. }\end{aligned}$$

Therefore,

3.4 $$\begin{aligned} d \mu (\xi ) = h(\bar{\xi })d \mu ^{c}(\xi ) \; \; \text{ and } \; \; h(\bar{\xi }) h(\xi )=1 \; \text{ for } \mu \text{-a.e. } \xi \in \mathbb {T}\text{. } \end{aligned}$$

which completes the proof. $\square$

The following proposition, originally explored in [16] for symmetric conjugations, relates a $C \in {\mathscr {C}}_{c}(U)$ with the associated spectral measure $E(\cdot )$ for *U*. Define $E^{c}(\cdot )$ on Borel subsets $\Omega$ of $\mathbb {T}$ by $E^{c}(\Omega ):= E(\Omega ^{*}).$ From this definition it follows that $\langle E^{c}(\Omega ) \textbf{x}, \textbf{y}\rangle =\mu _{\textbf{x}, \textbf{y}}^{c}(\Omega )$ for all $\textbf{x}, \textbf{y} \in {\mathcal {H}}$.

### Proposition 3.5

Let *C* be a conjugation on ${\mathcal {H}}$ and *U* be a unitary operator on ${\mathcal {H}}$ with associated spectral measure $E(\cdot )$. Then we have the following.

- $E^c(\cdot )$ is the associated spectral measure for $U^*$.
- $CE(\cdot )C$ is the spectral measure for $CU^*C$.
- $CUC = U^{*}$ if and only if $C E(\Omega ) C = E(\Omega )$ for all Borel subsets $\Omega$ of $\mathbb {T}$.
- $CUC = U$ if and only if $C E(\Omega )C = E^{c}(\Omega )$ for all Borel subsets $\Omega$ of $\mathbb {T}$.

### Proof

If $E(\cdot )$ is a spectral measure, one can check that $E^c(\cdot )$ and $CE(\cdot )C$ are also spectral measures. Since, for each pair $\textbf{x}, \textbf{y} \in {\mathcal {H}}$,

$$\begin{aligned} \langle U^* \textbf{x},\textbf{y}\rangle =\int \bar{\xi }\,d\langle E(\xi )\textbf{x},\textbf{y}\rangle =\int \xi \,d\langle E^c(\xi )\textbf{x},\textbf{y}\rangle , \end{aligned}$$

the uniqueness of the spectral measure for a unitary operator gives (a). In a similar way, (b) is a consequence of the computation

$$\begin{aligned} \langle CU^*C \textbf{x},\textbf{y}\rangle&=\langle C \textbf{y},U^*C \textbf{x}\rangle =\langle UC\textbf{y},C\textbf{x}\rangle \\&= \int \xi \,d\langle E(\xi )C\textbf{y},C\textbf{x}\rangle \\&=\int \xi \,d\langle \textbf{x},CE(\xi )C\textbf{y}\rangle =\int \xi \,d\langle CE(\xi )C\textbf{x},\textbf{y}\rangle . \end{aligned}$$

Note the use of (2.1) in the above calculation. To see (c), note that $CU^*C = U$ if and only if their spectral measures $CE(\cdot )C$ and $E(\cdot )$ coincide. Symmetrically in (d), $CU^*C$ equals to $U^*$ if and only if the spectral measures $CE(\cdot )C$ and $E^c(\cdot )$ coincide. $\quad \square$

As we will see in subsequent sections, the set ${\mathscr {C}}_c(U)$ is quite large and so an important step in understanding it is to decompose each $C \in {\mathscr {C}}_{c}(U)$ into more manageable pieces. This decomposition will involve various types of invariant subspaces. Recall that a (closed) subspace ${\mathcal {M}}$ of a Hilbert space ${\mathcal {H}}$ is *invariant* for an $A \in {\mathcal {B}}({\mathcal {H}})$ if $A {\mathcal {M}} \subseteq {\mathcal {M}}$; *reducing* if both $A {\mathcal {M}} \subseteq {\mathcal {M}}$ and $A^{*} {\mathcal {M}} \subseteq {\mathcal {M}}$; and *hyperinvariant* if $T {\mathcal {M}} \subseteq {\mathcal {M}}$ for every $T \in {\mathcal {B}}({\mathcal {H}})$ that commutes with *A*. We begin with a simple lemma whose proof follows from (2.1) and the fact that $C^2 = I$.

### Lemma 3.6

If *C* is a conjugation on ${\mathcal {H}}$ and ${\mathcal {M}}$ is a subspace of ${\mathcal {H}}$ such that $C {\mathcal {M}} \subseteq {\mathcal {M}}$, then $C {\mathcal {M}} = {\mathcal {M}}$ and $C{\mathcal {M}}^{\perp }={\mathcal {M}}^\perp$.

### Proposition 3.7

Let $U\in {\mathcal {B}}({\mathcal {H}})$ be a unitary operator with associated spectral measure $E(\cdot )$ and $\Omega \subseteq \mathbb {T}$ be a Borel set.

- If ${\Omega }^*=\Omega$ then for any $C \in {\mathscr {C}}_{c}(U)$, we have $C(E(\Omega ){\mathcal {H}}) = E(\Omega ){\mathcal {H}}$.
- If ${\mathscr {C}}_{c}(U) \not = \varnothing$ and $E(\Omega ){\mathcal {H}}$ is invariant for *C*, then $E(\Omega \setminus \Omega ^*)=0$.

### Proof

For the proof of (a), let $\textbf{x}\in E(\Omega ){\mathcal {H}}$ and $\textbf{y}\in (E(\Omega ){\mathcal {H}})^\perp$. By Proposition 3.5(d) we have

$$\begin{aligned} \langle C\textbf{x}, \textbf{y} \rangle = \langle CE(\Omega )\textbf{x}, \textbf{y} \rangle = \langle E(\Omega ^{*})C\textbf{x}, \textbf{y} \rangle = \langle C\textbf{x}, E(\Omega )\textbf{y} \rangle = \langle C \textbf{x}, \textbf{0}\rangle =0\end{aligned}$$

and thus $C\textbf{x}\in E(\Omega ){\mathcal {H}}$. Now apply Lemma 3.6.

For the proof of (b) let $\textbf{x}\in E(\Omega \setminus \Omega ^*){\mathcal {H}}$. From $E(\Omega )= E(\Omega ^{*}) \oplus E(\Omega {\setminus } \Omega ^{*})$, we can use Proposition 3.5(d) to see that

$$\begin{aligned} 0&=\Vert E(\Omega ^*)\textbf{x}\Vert =\Vert CE(\Omega )C \textbf{x}\Vert =\Vert E(\Omega )C\textbf{x}\Vert =\Vert C\textbf{x}\Vert =\Vert \textbf{x}\Vert . \end{aligned}$$

$$\square $$

For a unitary operator *U* on ${\mathcal {H}}$, one can show, as was done in [19], that for any $\mu \in M_{+}(\mathbb {T})$ the set

$$\begin{aligned} {\mathcal {H}}_\mu :=\{\textbf{x} \in {\mathcal {H}}: \mu _{\textbf{x}} \ll \mu \} \end{aligned}$$

is a reducing subspace of *U*. The space ${\mathcal {H}}_{\mu }$ was explored in [17, §65] as part of a general discussion of the multiplicity theory for unitary operators.

### Theorem 3.8

Let *U* be a unitary operator on ${\mathcal {H}}$, $E(\cdot )$ its associated spectral measure, $\mu \in M_{+}(\mathbb {T})$, and $C \in {\mathscr {C}}_{c}(U)$. Then we have the following.

- $C{\mathcal {H}}_\mu = {\mathcal {H}}_{\mu ^c}$ and $C{\mathcal {H}}_\mu ^{\perp }= {\mathcal {H}}_{\mu ^c}^\perp$, and thus
- $C = C_{\mu , \mu ^{c}} \oplus C^{'}_{\mu , \mu ^{c}}$, where $C_{\mu , \mu ^{c}}=C|_{{{\mathcal {H}}_\mu }}: {\mathcal {H}}_{\mu } \rightarrow {\mathcal {H}}_{\mu ^{c}}$ and $C^{'}_{\mu , \mu ^{c}}=C|_{{{\mathcal {H}}_\mu ^\perp }}: {\mathcal {H}}_{\mu }^{\perp } \rightarrow {\mathcal {H}}_{\mu ^c}^{\perp }$ are antilinear, onto, isometries.

### Proof

Let $\textbf{x}\in {\mathcal {H}}_\mu$. By Proposition 3.5(d), $CE(\cdot )C=E^c(\cdot )$ and thus

$$\begin{aligned} \langle E(\cdot ) C \textbf{x}, C \textbf{x}\rangle = \langle \textbf{x}, C E(\cdot ) C \textbf{x}\rangle = \langle \textbf{x}, E^c(\cdot ) \textbf{x}\rangle = \langle E^c(\cdot ) \textbf{x}, \textbf{x}\rangle . \end{aligned}$$

Since $\langle E(\cdot )\textbf{x},\textbf{x}\rangle \ll \mu$, it follows that $\langle E(\cdot ) C\textbf{x}, C\textbf{x}\rangle \ll \mu ^c$ and thus $C\textbf{x}\in {\mathcal {H}}_{\mu ^c}$. Similarly, $C{\mathcal {H}}_{\mu ^c}\subseteq {\mathcal {H}}_{\mu }$, thus $C{\mathcal {H}}_\mu = {\mathcal {H}}_{\mu ^c}$ and $C{\mathcal {H}}_\mu ^\perp = {\mathcal {H}}_{\mu ^c}^\perp$ (Lemma 3.6). $\square$

Recall [17, §48] the standard Boolean operations $\wedge$ and $\vee$ for $\mu _1, \mu _2 \in M_{+}(\mathbb {T})$ defined on Borel subsets $\Omega$ of $\mathbb {T}$ by

$$\begin{aligned}&(\mu _1 \vee \mu _2) (\Omega ):= \mu _1(\Omega ) + \mu _2(\Omega ); \\&(\mu _1 \wedge \mu _2)(\Omega ):= \inf \{\mu _1(\Omega \cap A) + \mu _2(\Omega \setminus A): A \text { is a Borel set}\}. \end{aligned}$$

For a unitary *U*, there exists a *scalar spectral measure* $\nu$, meaning that $\nu (\Delta ) = 0$ if and only if $E(\Delta ) = 0$ [5, p. 293] (also see the discussion in Theorem 3.8 in [19]). For $\nu _1, \nu _2 \in M_{+}(\mathbb {T})$ it was shown in [19, Prop. 3.10] that ${\mathcal {H}}_{\nu _1}\subseteq {\mathcal {H}}_{\nu _2}$ if and only if $\nu _1\wedge \mu \ll \nu _2\wedge \mu .$

### Corollary 3.9

Let *U* be a unitary operator on ${\mathcal {H}}$ and $\nu$ be any scalar spectral measure for *U*. Suppose that $\mu \in M_{+}(\mathbb {T})$ satisfies $\mu ^{c} \wedge \nu \ll \mu \wedge \nu$. If $C \in {\mathscr {C}}_{c}(U)$, we have the following.

- $$C {\mathcal {H}}_{\mu } = {\mathcal {H}}_{\mu }$$ and $$C {\mathcal {H}}_{\mu }^{\perp } = {\mathcal {H}}_{\mu }^{\perp }$$.
- $$C = C_{\mu } \oplus C_{\mu }^{\perp }$$, where $$C_{\mu }:= C|_{{\mathcal {H}}_{\mu }}$$ and $$C_{\mu }^{\perp } = C|_{{\mathcal {H}}_{\mu }^{\perp }}$$.
- $$C_{\mu } \in {\mathscr {C}}_{c}(U|_{{\mathcal {H}}_{\mu }})$$ and $$C_{\mu }^{\perp } \in {\mathscr {C}}_{c}(U|_{{\mathcal {H}}_{\mu }^{\perp }})$$.

### Corollary 3.10

Let *U* be a unitary on ${\mathcal {H}}$ and $\nu$ be any scalar spectral measure for *U*. Fix a $\mu \in M_+(\mathbb {T})$. If $C{\mathcal {H}}_\mu \subseteq {\mathcal {H}}_\mu$ for some $C\in {\mathscr {C}}_c(U)$ then $\mu ^c\wedge \nu \ll \mu \wedge \nu$.

### Proof

By Theorem 3.8 we have $C{\mathcal {H}}_\mu ={\mathcal {H}}_{\mu ^c}\subseteq {\mathcal {H}}_\mu$. Thus, by [19, Prop. 3.11], we obtain $\mu ^c\wedge \nu \ll \mu \wedge \nu$. $\square$

Since a unitary operator is normal, we see that $\ker (U-\alpha I)=\ker (U^*-\bar{\alpha }I)$ i.e., ${\mathcal {H}}_{\delta _\alpha }={\mathcal {H}}_{{\delta _{\alpha }^c}}$, where $\delta _{\alpha }$ denotes an atomic measure with atom at $\alpha \in \mathbb {T}$. This gives us the following corollary.

### Corollary 3.11

Let *U* be a unitary operator on ${\mathcal {H}}$ and $C \in {\mathscr {C}}_{c}(U)$. Let $\alpha \in {\mathbb {T}}$ be an eigenvalue for *U*. Then $C=C_{\delta _\alpha }\oplus C_{\delta _\alpha }^\perp ,$ where $C_{\delta _{\alpha }} = C|_{{\mathcal {H}}_{\delta _{\alpha }}}$ and $C_{\delta _{\alpha }}^{\perp } = C|_{{\mathcal {H}}_{\delta _{\alpha }}^{\perp }}$ are conjugations on $\ker (U-\alpha I)$ and $\ker (U-\alpha I)^\perp$, respectively.

## Natural conjugations on vector valued $L^2$ spaces

This section provides a model for conjugations on vector valued Lebesgue spaces and will be useful in our description of ${\mathscr {C}}_c(U)$ in Theorem 5.3.

For a Hilbert space ${\mathcal {H}}$ with norm $\Vert \cdot \Vert _{{\mathcal {H}}}$ and a $\mu \in M_{+}(\mathbb {T})$, consider the set ${\mathscr {L}}^{0}(\mu , {\mathcal {H}})$ of ${\mathcal {H}}$-valued $\mu$-measurable functions ${\varvec{f}}$ on $\mathbb {T}$ and the set

$$\begin{aligned} {\mathscr {L}}^2(\mu , {\mathcal {H}}):= \Big \{{\varvec{f}}\in {\mathscr {L}}^{0}(\mu , {\mathcal {H}}): \Vert {\varvec{f}}\Vert _{L^2(\mu . {\mathcal {H}})}:= \Big (\int _{\mathbb {T}} \Vert {\varvec{f}}(\xi )\Vert ^{2}_{{\mathcal {H}}}d\mu (\xi )\Big )^{\frac{1}{2}} < \infty \Big \}. \end{aligned}$$

Also consider ${\mathscr {L}}^{\infty }(\mu , {\mathcal {B}}({\mathcal {H}}))$, the $\mu$-essentially bounded ${\mathcal {B}}({\mathcal {H}})$-valued functions ${{\varvec{U}}}$ on $\mathbb {T}$. For ${{\varvec{U}}}\in {\mathscr {L}}^{\infty }(\mu , {\mathcal {B}}({\mathcal {H}}))$, define the multiplication operator ${{\varvec{M}}}_{{{\varvec{U}}}}$ on ${\mathscr {L}}^2(\mu , {\mathcal {H}})$ by

$$\begin{aligned} ({{\varvec{M}}}_{{{\varvec{U}}}}{\varvec{f}})(\xi )={{\varvec{U}}}(\xi ){\varvec{f}}(\xi ) \end{aligned}$$

for ${\varvec{f}}\in {\mathscr {L}}^2(\mu ,{\mathcal {H}})$ and $\mu$-a.e.  $\xi \in \mathbb {T}$. Clearly ${{\varvec{M}}}_{{{\varvec{U}}}}\in {\mathcal {B}}({\mathscr {L}}^2(\mu ,{\mathcal {H}}))$. If we use the notation ${{\varvec{U}}}^{*}(\xi ) = {{\varvec{U}}}(\xi )^{*}$, one can verify that

4.1 $$\begin{aligned} {{\varvec{M}}}_{{{\varvec{U}}}}^{*} = {{\varvec{M}}}_{{{\varvec{U}}}^{*}}. \end{aligned}$$

We will use $L^{\infty }(\mu ):= {\mathscr {L}}^{\infty }(\mu , \mathbb {C})$ to denote the scalar valued $\mu$-essentially bounded functions on $\mathbb {T}$. For ease of notation, we will write ${{\varvec{M}}}_{\varphi }$, when $\varphi \in L^{\infty }(\mu )$, instead of the more cumbersome ${{\varvec{M}}}_{\varphi {\varvec{I}}_{{\mathcal {H}}}}$, that is,

$$\begin{aligned} ({{\varvec{M}}}_\varphi {\varvec{f}})(\xi )=({{\varvec{M}}}_{\varphi {\varvec{I}}_{\mathcal {H}}}{\varvec{f}})(\xi )=\varphi (\xi ){\varvec{f}}(\xi ) \end{aligned}$$

for ${\varvec{f}}\in {\mathscr {L}}^2(\mu , {\mathcal {H}})$ and $\mu$-a.e.  $\xi \in \mathbb {T}$. The case when $\varphi (\xi ) = \xi$ will play an prominent role in this paper in which case we have the vector-valued bilateral shift ${{\varvec{M}}}_{\xi }$ on ${\mathscr {L}}^2(\mu , {\mathcal {H}})$.

Recall from Sect. 2 that ${\mathscr {A}}\!{\mathcal {B}}({\mathcal {H}})$ denotes the space of all bounded antilinear operators on ${\mathcal {H}}$. We define ${\mathscr {L}}^{\infty }(\mu , {\mathscr {A}}\!{\mathcal {B}}({\mathcal {H}}))$ to be the space of all $\mu$-essentially bounded and ${\mathscr {A}}\!{\mathcal {B}}({\mathcal {H}})$-valued Borel functions on $\mathbb {T}$. Similarly as above, for ${{\varvec{C}}}\in {\mathscr {L}}^{\infty }(\mu , {\mathscr {A}}\!{\mathcal {B}}({\mathcal {H}}))$, define

$$\begin{aligned} ({{\varvec{A}}}_{{\varvec{C}}}{\varvec{f}})(\xi )={{\varvec{C}}}(\xi ){\varvec{f}}(\xi ) \end{aligned}$$

for ${\varvec{f}}\in {\mathscr {L}}^2(\mu ,{\mathcal {H}})$ and $\mu$-a.e.  $\xi \in \mathbb {T}$. One can check that ${{\varvec{A}}}_{{\varvec{C}}}\in {\mathscr {A}}\!{\mathcal {B}}({\mathscr {L}}^2(\mu ,{\mathcal {H}}))$.

For any conjugation *J* on ${\mathcal {H}}$, define the conjugation ${\varvec{J}}$ on ${\mathscr {L}}^2(\mu ,{\mathcal {H}})$ by

$$\begin{aligned} ({\varvec{J}}{\varvec{f}})(\xi )=J({\varvec{f}}(\xi )), \; \; {\varvec{f}}\in {\mathscr {L}}^2(\mu ,{\mathcal {H}}). \end{aligned}$$

Notice that $$ {\varvec{J}}{{\varvec{M}}}_\xi {\varvec{J}}={{\varvec{M}}}_{\bar{\xi }} $$ [19].

We now focus our attention on the scalar valued $L^2(\mu )$ space and the set ${\mathscr {C}}_{c}(M_{\xi })$. This next result shows that when ${\mathscr {C}}_{c}(M_{\xi }) \not = \varnothing$, there must be some restrictions on $\mu$. The set ${\mathscr {C}}_{c}(M_{\xi })$ was explored in [4] when $\mu = m$.

### Proposition 4.2

Let $\mu \in M_{+}(\mathbb {T})$ and *C* be a conjugation on $L^2(\mu )$ such that $C M_\xi =M_\xi C$. Then $\mu ^c \ll \mu$ (and hence $\mu \ll \mu ^{c}$ by Proposition 3.3).

### Proof

From (2.5), the identity $C M_ \xi C=M_\xi$ implies that $CM_{\bar{\xi }}C=M_{\bar{\xi }}$. For any trigonometric polynomial $p(\xi )$ define $p^{\#}(\xi ):= \overline{p(\overline{\xi })}.$ The above (and the antilinearity of *C*) shows that $CM_{p}C=M_{p^{\#}}.$ Therefore, by the weak-$*$ density of the trigonometric polynomials in $L^{\infty }(\mu )$, we obtain

4.3 $$\begin{aligned} CM_{\varphi }C=M_{{\varphi }^{\#}} \; \text {for any } \varphi \in L^\infty (\mu ),\end{aligned}$$

where ${\varphi }^{\#}(\xi )=\overline{\varphi (\overline{\xi })}.$ If $\mu ^c \not \ll \mu$, then there is a Borel set $\Omega \subseteq \mathbb {T}$ such that $\mu (\Omega )\not = 0$ but $\mu ^c(\Omega )= 0$. But, (4.3) yields a contradiction with $\varphi =\chi _\Omega$, since $M_{\chi _{\Omega ^*}}=0$ but $CM_{\chi _\Omega }C$ is not. $\square$

Now let us focus on the situation when $\mu ^c\ll \mu$. In this case we also have that $\mu \ll \mu ^c$ (Proposition 3.3). For ${\varvec{f}}\in {\mathscr {L}}^2(\mu ,{\mathcal {H}})$ and ${{\varvec{U}}}\in {\mathscr {L}}^{\infty }(\mu , {\mathcal {B}}({\mathcal {H}}))$, it makes sense to write ${\varvec{f}}(\bar{\xi })$ or ${{\varvec{U}}}(\bar{\xi })$ and define

4.4 $$\begin{aligned} {{\varvec{U}}}^\#(\xi ):={{\varvec{U}}}^*(\bar{\xi })={{\varvec{U}}}(\bar{\xi })^*. \end{aligned}$$

### Proposition 4.5

Let $\mu \in M_{+}(\mathbb {T})$ such that $\mu ^c \ll \mu$ and let $h = d \mu ^{c}/d \mu$. For a Hilbert space ${\mathcal {H}}$, a conjugation *J* on ${\mathcal {H}}$, and ${\varvec{f}}\in {\mathscr {L}}^2(\mu , {\mathcal {H}})$, define

4.6 $$\begin{aligned} ({\varvec{J}}^{\#}{\varvec{f}})(\xi )= (h(\xi ))^{\frac{1}{2}}J({\varvec{f}}(\bar{\xi })) \end{aligned}$$

for $\mu$-a.e.  $\xi \in \mathbb {T}$. Then we have the following.

- ${\varvec{J}}^{\#}$ is a conjugation on ${\mathscr {L}}^2(\mu ,{\mathcal {H}})$;
- $$ {\varvec{J}}^{\#}{{\varvec{M}}}_\xi {\varvec{J}}^{\#}={{\varvec{M}}}_\xi $$.

### Proof

As discussed in Proposition 3.3, $\mu \ll \mu ^c$ and $d\mu ^c=h^{\#}\,d\mu$ with $h^\#(\xi )\, h(\xi )=h(\bar{\xi })h(\xi )=1$ for $\mu$ a.e.  $\xi \in \mathbb {T}$.

Since *J* is antilinear on ${\mathcal {H}}$, one sees that ${\varvec{J}}^{\#}$ is antilinear on ${\mathscr {L}}^2(\mu , {\mathcal {H}})$. Moreover, for ${\varvec{f}}\in {\mathscr {L}}^2(\mu ,{\mathcal {H}})$ we have

$$\begin{aligned} \Vert {\varvec{J}}^{\#}{\varvec{f}}\Vert _{{\mathscr {L}}^2(\mu , {\mathcal {H}})}^{2}&=\int \Vert h(\xi )^{\frac{1}{2}}\,J({\varvec{f}}(\bar{\xi }))\Vert _{{\mathcal {H}}}^2d\mu (\xi )\\&= \int \Vert J({\varvec{f}}(\bar{\xi }))\Vert _{{\mathcal {H}}}^2\, h(\xi )\,d\mu (\xi )\\&= \int \Vert {\varvec{f}}( \xi )\Vert _{{\mathcal {H}}}^2\, h(\bar{\xi })\,d\mu (\bar{\xi }) = \int \Vert {\varvec{f}}(\xi )\Vert _{{\mathcal {H}}}^2\,d\mu ( \xi ) =\Vert {\varvec{f}}\Vert _{{\mathscr {L}}^2(\mu , {\mathcal {H}})}^2. \end{aligned}$$

Note the use of (3.4) above. Thus, ${\varvec{J}}^{\#}$ is isometric on ${\mathscr {L}}^2(\mu , {\mathcal {H}})$.

Next we show that $({\varvec{J}}^{\#})^2=I$. Indeed, for each ${\varvec{f}}\in {\mathscr {L}}^2(\mu , {\mathcal {H}})$,

$$\begin{aligned} ({\varvec{J}}^{\#} {\varvec{J}}^{\#}{\varvec{f}}) (\xi )&=h(\xi )^{\frac{1}{2}}\, J(({\varvec{J}}^{\#}{\varvec{f}})(\bar{\xi }))\\&= h( \xi )^{\frac{1}{2}}\, J\Big ((h(\bar{\xi }))^{\frac{1}{2}}\, J({\varvec{f}}( \xi ))\Big ) \\&= (h( \xi ) h(\bar{\xi })^{\frac{1}{2}}\, J( J({\varvec{f}}( \xi ))) ={\varvec{f}}(\xi ). \end{aligned}$$

Again, note the use of (3.4) above. Therefore, ${\varvec{J}}^{\#}$ is a conjugation. To prove (b), observe that for each ${\varvec{f}}\in {\mathscr {L}}^2(\mu , {\mathcal {H}})$ we have

$$\begin{aligned} ({\varvec{J}}^{\#}{{\varvec{M}}}_\xi {\varvec{f}})(\xi )&= {\varvec{J}}^{\#}({{\varvec{M}}}_\xi {\varvec{f}})(\xi ) = h( \xi )^{\frac{1}{2}}\, J(({{\varvec{M}}}_\xi {\varvec{f}})(\bar{\xi }))\\&=h( \xi )^{\frac{1}{2}}\, J(\bar{\xi }{\varvec{f}}(\bar{\xi })) = \xi h( \xi )^{\frac{1}{2}}\, J({\varvec{f}}(\bar{\xi }))\\&= \xi ({\varvec{J}}^{\#}{\varvec{f}})(\xi ) = ({{\varvec{M}}}_\xi {\varvec{J}}^{\#}){\varvec{f}}(\xi ). \end{aligned}$$

$$\square $$

### Remark 4.7

If $\mu = m$, Lebesgue measure on ${\mathbb {T}}$, then $m=m^c$ and $h\equiv 1$ and the conjugation (4.6) coincides with the one considered in [4].

A special case worth pointing out is the scalar case ${\mathcal {H}}={\mathbb {C}}$.

### Corollary 4.8

Let $\mu \in M_{+}(\mathbb {T})$ such that $\mu ^c\ll \mu$. Let $h = d\mu ^{c}/d \mu$ and define

4.9 $$\begin{aligned} (J^{\#} f)(\xi )= h( \xi )^{\frac{1}{2}}\ \overline{f(\bar{\xi })}, \quad f \in L^2(\mu ). \end{aligned}$$

Then $J^{\#}$ is a conjugation on $L^2(\mu )$ and $J^{\#} M_\xi J^{\#}= M_\xi$.

In particular, observe that $\mu ^{c} \ll \mu \Longrightarrow {\mathscr {C}}_{c}(M_{\xi }) \not = \varnothing$.

The following echos a result from [4, Proposition 4.2]. Recall the notation from (4.4).

### Proposition 4.10

Let *J* be a conjugation on ${\mathcal {H}}$, ${\varvec{J}}^\#$ be defined by (4.6), and let ${{\varvec{U}}}\in {\mathscr {L}}^{\infty }(\mu , {\mathcal {B}}({\mathcal {H}}))$ be a unitary operator-valued function. Then we have the following.

- $${\varvec{J}}^\#{{\varvec{M}}}_{{\varvec{U}}}{\varvec{J}}^{\#}={\varvec{J}}{{\varvec{M}}}_{({{\varvec{U}}}^\#)^*} {\varvec{J}}$$;
- ${{\varvec{M}}}_{{{\varvec{U}}}}{\varvec{J}}^{\#}$ is a conjugation on ${\mathscr {L}}^2(\mu , {\mathcal {H}})$ if and only if $J{{\varvec{U}}}( \xi )J={{\varvec{U}}}^\#( \xi )={{\varvec{U}}}^*(\bar{\xi })$ for $\mu$-a.e.  $\xi \in \mathbb {T}$;
- If ${{\varvec{M}}}_{{{\varvec{U}}}}{\varvec{J}}^{\#}$ is a conjugation on ${\mathscr {L}}^2(\mu , {\mathcal {H}})$ then ${{\varvec{M}}}_{{{\varvec{U}}}}{\varvec{J}}^{\#}={\varvec{J}}^{\#}{{\varvec{M}}}_{{{\varvec{U}}}^*}$;
- $$\big ({{\varvec{M}}}_{{\varvec{U}}}{\varvec{J}}^{\#}\big ){{\varvec{M}}}_\xi \big ({{\varvec{M}}}_{{\varvec{U}}}{\varvec{J}}^{\#}\big )={{\varvec{M}}}_{\xi }$$.

### Proof

For every ${\varvec{f}}\in {\mathscr {L}}^2(\mu , {\mathcal {H}})$, observe that for $\mu$-a.e.  $\xi \in \mathbb {T}$ we have

$$\begin{aligned} ({\varvec{J}}^\#{{\varvec{M}}}_{{\varvec{U}}}{\varvec{J}}^{\#} {\varvec{f}})(\xi )&=h( \xi )^{\frac{1}{2}}J\big (({{\varvec{M}}}_{{\varvec{U}}}{\varvec{J}}^{\#} {\varvec{f}})(\bar{\xi })\big )\\&=h( \xi )^{\frac{1}{2}}\,J\big ({{\varvec{U}}}(\bar{\xi })\big ({\varvec{J}}^{\#} {\varvec{f}}\big )(\bar{\xi })\big )\\&=h( \xi )^{\frac{1}{2}}\,J\big ({{\varvec{U}}}(\bar{\xi })h(\bar{\xi })^{\frac{1}{2}} J ({\varvec{f}}(\xi )\big ))\\&=(h( \xi )h(\bar{\xi }))^{\frac{1}{2}}\,J({{\varvec{U}}}(\bar{\xi }) J({\varvec{f}}( \xi )))\\&=J{{\varvec{U}}}(\bar{\xi }) J({\varvec{f}}( \xi )) =J(({{\varvec{U}}}^\#(\xi ))^* J({\varvec{f}}( \xi )))\\&=({\varvec{J}}({{\varvec{U}}}^\#)^* {\varvec{J}}{\varvec{f}})( \xi ). \end{aligned}$$

Note the use of (4.4) above. This proves (a).

Note that ${{\varvec{M}}}_{{\varvec{U}}}{\varvec{J}}^{\#}$ is antilinear and isometric on ${\mathscr {L}}^2(\mu , {\mathcal {H}})$. To prove that ${{\varvec{M}}}_{{{\varvec{U}}}} {\varvec{J}}^{\#}$ is a conjugation (and thus complete the proof of (b)), Lemma 2.7 says that we just need to check the identity ${\varvec{J}}^{\#}{{\varvec{M}}}_{{\varvec{U}}}{\varvec{J}}^{\#} = {{\varvec{M}}}_{{{\varvec{U}}}}^{*}.$ By (a) this is equivalent to $J{{\varvec{U}}}(\bar{\xi })J={{\varvec{U}}}^*(\xi )$ since, by (4.1), $({{\varvec{M}}}_{{\varvec{U}}}^*{\varvec{f}})(\xi )={{\varvec{U}}}^*(\xi ){\varvec{f}}(\xi )$.

Statement (c) follows from the fact that ${{\varvec{M}}}_{{{\varvec{U}}}} {\varvec{J}}^{\#}$ is a conjugation on ${\mathscr {L}}^2(\mu , {\mathcal {H}})$, and so $({{\varvec{M}}}_{{{\varvec{U}}}} {\varvec{J}}^{\#})( {{\varvec{M}}}_{{{\varvec{U}}}} {\varvec{J}}^{\#}) = I$, along with the fact ${{\varvec{M}}}_{{{\varvec{U}}}} {{\varvec{M}}}_{{{\varvec{U}}}^{*}} = {{\varvec{M}}}_{{{\varvec{U}}}^{*}} {{\varvec{M}}}_{{{\varvec{U}}}} = I$ (since ${{\varvec{U}}}(\xi )$ is unitary for $\mu$-a.e.  $\xi \in \mathbb {T}$).

To see (d), observe that for any ${\varvec{f}}\in {\mathscr {L}}^2(\mu , {\mathcal {H}})$,

$$\begin{aligned} ({{\varvec{M}}}_{{\varvec{U}}}{\varvec{J}}^{\#}{{\varvec{M}}}_\xi {\varvec{f}})(\xi )&= {{\varvec{U}}}(\xi ){\varvec{J}}^{\#}({{\varvec{M}}}_\xi {\varvec{f}})(\xi )\\&= {{\varvec{U}}}(\xi )h( \xi )^{\frac{1}{2}}\, J(({{\varvec{M}}}_\xi {\varvec{f}})(\bar{\xi }))\\&=h( \xi )^{\frac{1}{2}}\,{{\varvec{U}}}(\xi ) J(\bar{\xi }{\varvec{f}}(\bar{\xi })) =\xi h( \xi )^{\frac{1}{2}}\, {{\varvec{U}}}(\xi )J({\varvec{f}}(\bar{\xi })) \end{aligned}$$

while

$$\begin{aligned} ({{\varvec{M}}}_\xi {{\varvec{M}}}_{{\varvec{U}}}{\varvec{J}}^{\#}{\varvec{f}})(\xi )&= \xi ({{\varvec{M}}}_{{\varvec{U}}}{\varvec{J}}^{\#}{\varvec{f}})(\xi ) = \xi {{\varvec{U}}}(\xi )({\varvec{J}}^{\#}{\varvec{f}})(\xi ) \\&=\xi {{\varvec{U}}}(\xi )h( \xi )^{\frac{1}{2}}\, J({\varvec{f}}(\bar{\xi })) = \xi h( \xi )^{\frac{1}{2}}\, {{\varvec{U}}}(\xi )J({\varvec{f}}(\bar{\xi })), \end{aligned}$$

which completes the proof of (d). $\square$

## Conjugations via the spectral theorem

In this section we use the multiplicity theory for unitary operators [5, 17] to describe ${\mathscr {C}}_{c}(U)$. We also prove that ${\mathscr {C}}_{c}(U) \not = \varnothing$ if and only if $U \cong U^{*}$ (thus establishing the converse to Lemma 2.9). We begin with a statement of the spectral multiplicity theory from [5, p.  307, Ch. IX, Theorem 10.20]. Recall the statement of the multiplicity version of the spectral theorem from (1.3).

### Remark 5.1

Let *U* be a unitary operator with a spectral measure $E(\cdot )$. As previously observed in Proposition 3.5(a), $E^c(\cdot )$ is a spectral measure for $U^*$. In [19, Theorem 8.1], the measures $\mu _\infty ,\mu _1,\mu _2,\dots$ from (1.3) were constructed using the spectral measure $E(\cdot )$. Therefore, the appropriate measures for operator $U^*$ are $\mu ^c_\infty ,\mu ^c_1,\mu ^c_2,\dots$.

### Lemma 5.2

Let *U* be a unitary operator on ${\mathcal {H}}$ with the multiplicity representation of *U* given by the mutually singular measures $\mu _\infty ,\mu _1,\mu _2,\dots$ as in (1.3). If ${\mathscr {C}}_{c}(U) \not = \varnothing$, then $\mu _i^c\ll \mu _i$ for all $i=\infty ,1,2,\dots$.

### Proof

Lemma 2.9 says that if ${\mathscr {C}}_{c}(U) \not = \varnothing$, then $U \cong U^{*}$. Hence, by Remark 5.1 and [5, p. 305, Theorem IX 10.16], the measures $\mu _i$ and $\mu ^c_i$ are mutually absolutely continuous for all $i=\infty ,1,2,\dots$. $\square$

We now arrive at the description of ${\mathscr {C}}_{c}(U)$ in terms of the parameters of the spectral theorem.

### Theorem 5.3

Let *U* be a unitary operator and *C* be a conjugation on ${\mathcal {H}}$. With the notation as in (1.3), assuming that $\mu _i^c\ll \mu _i$ for $i=\infty ,1,2,\dots$, the following are equivalent

- $$C \in {\mathscr {C}}_{c}(U)$$;
- For each $i=\infty ,1,2,\dots$, there are conjugations ${{\varvec{C}}}^{i}\in {\mathscr {A}}\! {\mathcal {B}}(({\mathscr {L}}^{2}(\mu _i,{\mathcal {H}}_i))$ such that ${{\varvec{M}}}_\xi ^{(i)}$ is ${{\varvec{C}}}^{i}$–commuting and $C={{\mathcal {I}}}^{*}\Big (\bigoplus {{\varvec{C}}}^i\Big ){\mathcal {I}};$
- For each $i=\infty ,1,2,\dots$ and any conjugation $J^{(i)}$ on ${\mathcal {H}}_i$, there is a unitary operator-valued function ${{\varvec{U}}}^{(i)}\in {\mathscr {L}}^{\infty }(\mu _i,{\mathcal {B}}({\mathcal {H}}_i))$ such that
  $$\begin{aligned}{} & {} J^{(i)}{{\varvec{U}}}^{(i)}(\xi )J^{(i)}={{\varvec{U}}}^{(i)}(\xi )^{\#} \; \text{ for } \mu _i \text{ a.e. } \xi \in \mathbb {T} \text{ and } \\{} & {} C={{\mathcal {I}}}^{*}\Big (\bigoplus {{\varvec{U}}}^{(i)}{{\varvec{J}}^{\#}}^{(i)}\Big ){\mathcal {I}}={{\mathcal {I}}}^{*}\Big (\bigoplus {{\varvec{U}}}^{(i)}\Big )\Big (\bigoplus {{\varvec{J}}^{\#}}^{(i)}\Big ){\mathcal {I}}. \end{aligned}$$

### Proof

To show (a) $\Longrightarrow$ (c), let $\widetilde{{{\varvec{M}}}}_\xi := {\mathcal {I}}U{\mathcal {I}}^{*}\in {\mathcal {B}}({{\varvec{L}}}^2_{{\mathcal {H}}})$ and define the conjugation $\widetilde{\!{{\varvec{C}}}}={\mathcal {I}}C{\mathcal {I}}^{*}$ (note the use of Lemma 2.3). Then $\widetilde{\!{{\varvec{C}}}}\widetilde{{{\varvec{M}}}}_\xi \,\widetilde{\!{{\varvec{C}}}}=\widetilde{{{\varvec{M}}}}_{ \xi }.$

Let $J^{(i)}$ be any a conjugation on ${\mathcal {H}}_i$. Since $\mu ^c_i\ll \mu _i$ for $i=\infty ,1,2,\dots$, let $h_i = d \mu ^{c}_{i}/d \mu _{i}$ and define the map ${{\varvec{J}}^{\#}}^{(i)}$ on ${\mathscr {L}}^2(\mu _i,{\mathcal {H}}_i)$ by

$$\begin{aligned} ({{\varvec{J}}^{\#}}^{(i)}{\varvec{f}}_i)(\xi )= h_i( \xi )^{\frac{1}{2}}\, J^{(i)}({\varvec{f}}_i(\bar{\xi })) \end{aligned}$$

for $\mu _i$-a.e.  $\xi \in \mathbb {T}$ and ${\varvec{f}}_i\in {\mathscr {L}}^2(\mu _i,{\mathcal {H}}_i)$. By Proposition 4.5, each of the above maps defines a conjugation on ${\mathscr {L}}^2(\mu _i, {\mathcal {H}}_{i})$ which satisfies

$$\begin{aligned} {{\varvec{J}}^{\#}}^{(i)} {{\varvec{M}}}_\xi ^{(i)} {{\varvec{J}}^{\#}}^{(i)}={{\varvec{M}}}_\xi ^{(i)}. \end{aligned}$$

Use these conjugations to define the conjugation $\widetilde{\!{\varvec{J}}\,}^{\#}=\bigoplus {{\varvec{J}}^{\#}}^{(i)}$ on ${{\varvec{L}}}^2_{{\mathcal {H}}}$ and observe that

$$\begin{aligned} \widetilde{\!{\varvec{J}}\,}^{\#}\,\widetilde{{{\varvec{M}}}}_\xi \,\widetilde{\!{\varvec{J}}\,}^{\#}=\widetilde{{{\varvec{M}}}}_{ \xi }. \end{aligned}$$

Moreover,

$$\begin{aligned} \widetilde{{{\varvec{M}}}}_\xi \ \widetilde{\!{{\varvec{C}}}}\ \widetilde{\!{\varvec{J}}\,}^{\#}=\widetilde{\!{{\varvec{C}}}}\ \widetilde{{{\varvec{M}}}}_{\xi }\ \widetilde{\!{\varvec{J}}\,}^{\#}=\widetilde{\!{{\varvec{C}}}}\ \widetilde{\!{\varvec{J}}\,}^{\#}\ \widetilde{{{\varvec{M}}}}_\xi . \end{aligned}$$

The spectral theorem applied to $\widetilde{{{\varvec{M}}}_{\xi }}$ also yields the commutant [5, p.  307, Theorem 10.20], namely there are

$$\begin{aligned} {{\varvec{U}}}^{(i)}\in {\mathscr {L}}^{\infty }(\mu _i,{\mathcal {B}}({\mathcal {H}}_i)), \quad i=\infty ,1,2,\dots , \end{aligned}$$

such that

$$\begin{aligned} \widetilde{\!{{\varvec{C}}}}\, \widetilde{\!{\varvec{J}}\,}^{\#}=\bigoplus \widetilde{{{\varvec{M}}}}_{{{\varvec{U}}}^{(i)}}=\widetilde{{{\varvec{M}}}}_{{{\varvec{U}}}^{(\infty )}}\oplus \widetilde{{{\varvec{M}}}}_{{{\varvec{U}}}^{(1)}} \oplus \widetilde{{{\varvec{M}}}}_{{{\varvec{U}}}^{(2)}} \oplus \cdots . \end{aligned}$$

Since $\widetilde{\!{{\varvec{C}}}}\, \widetilde{\!{\varvec{J}}\,}^{\#}$ is unitary, it follows that $\widetilde{{{\varvec{M}}}}_{{{\varvec{U}}}^{(i)}}$ is also unitary and consequently ${{\varvec{U}}}^{(i)}$ is a operator-valued function such that ${{\varvec{U}}}^{(i)}(\xi )$ is unitary for $\mu _i$ a.e.  $\xi \in \mathbb {T}$. Therefore,

$$\begin{aligned} \widetilde{\!{{\varvec{C}}}}=\Big (\bigoplus {{\varvec{M}}}_{{{\varvec{U}}}^{(i)}}\Big )\Big (\bigoplus {{\varvec{J}}^{\#}}^{(i)}\Big )=\bigoplus {{\varvec{M}}}_{{{\varvec{U}}}^{(i)}}{{\varvec{J}}^{\#}}^{(i)}. \end{aligned}$$

Since $\widetilde{\!{{\varvec{C}}}}|_{{\mathscr {L}}^2(\mu _i,{\mathcal {H}}_i)}$ is a conjugation, it follows that

$$\begin{aligned} J^{(i)}{{\varvec{U}}}^{(i)}(\xi )J^{(i)}= ({{\varvec{U}}}^{(i)}( \xi ))^\# \end{aligned}$$

for $\mu _i$-a.e.  $\xi \in \mathbb {T}$ (Proposition 4.10). This completes the proof of (a) $\Longrightarrow$ (c).

To prove (c) $\Longrightarrow$ (b), it is enough to take ${{\varvec{C}}}^{(i)}={{\varvec{M}}}_{{{\varvec{U}}}^{(i)}}{{\varvec{J}}^{\#}}^{(i)}$. The remaining implication (b) $\Longrightarrow$ (a) is trivial. $\square$

The following yields the converse of Lemma 2.9 and thus completes the criterion as to when ${\mathscr {C}}_{c}(U) \not = \varnothing$.

### Corollary 5.4

If *U* is a unitary operator on ${\mathcal {H}}$ such that $U \cong U^{*}$, then there is conjugation *C* on ${\mathcal {H}}$ such that $C U C = U$.

### Proof

If $U \cong U^{*}$, then, as in the proof of Lemma 5.2, the measures $\mu _i$ and $\mu ^c_i$ are mutually absolutely continuous for all $i=\infty ,1,2,\dots$. Now invoke Theorem  5.3 with any conjugation $J^{(i)}$ on ${\mathcal {H}}_j$ (and ${{\varvec{U}}}^{(i)}=I_{{\mathcal {H}}_i}$) and observe that the conjugation $C={\mathcal {I}}^{*}{\varvec{J}}^{\#} {\mathcal {I}}={\mathcal {I}}^{*}\bigoplus {{\varvec{J}}^{\#}}^{(i)} {\mathcal {I}}$ commutes with *U*. $\square$

## Examples

In this section we use our results to give concrete descriptions of ${\mathscr {C}}_{c}(U)$ when *U* is a unitary matrix, multiplication by an inner function on $L^2(m)$, the Fourier transform, and the Hilbert transform.

### Unitary matrices

For an $n \times n$ unitary matrix *U*, the condition as to when ${\mathscr {C}}_{c}(U)$ is nonempty, along with the description of ${\mathscr {C}}_{c}(U)$, can certainly be derived from Theorem 5.3. However, we give a simple proof using basic linear algebra. We begin with the following result from [15, Lemma 3.2].

#### Proposition 6.1

A mapping *C* on $\mathbb {C}^n$ is a conjugation if and only if $C = V J$, where *V* is an $n \times n$ unitary matrix with $V^{t} = V$ and *J* is the conjugation on $\mathbb {C}^n$ defined by

6.2 $$\begin{aligned} J[x_1\; x_2\; \cdots \; x_n]^{t} = [\overline{x_1}\; \overline{x_2}\; \cdots \; \overline{x_n}]^{t}, \end{aligned}$$

i.e., $$C[x_1 \; x_2 \; \cdots \; x_n]^{t} = V [\overline{x_1} \; \overline{x_2} \; \cdots \; \overline{x_n}]^{t}.$$

The spectral theorem for unitary matrices says that if $U \cong U^{*}$, then *U* is unitarily equivalent to

6.3 $$\begin{aligned} U' = \begin{bmatrix} \begin{bmatrix} \xi _1 I_{n_1} &{} \\ &{} \overline{\xi _1} I_{n_1} \end{bmatrix} &{} &{} \\ &{} \ddots &{} &{} \\ &{} &{} &{} \begin{bmatrix} \xi _d I_{n_d} &{} \\ &{} \overline{\xi _d} I_{n_d} \end{bmatrix}\\ &{} &{} &{} &{} \begin{bmatrix} I_{\ell } &{} \\ &{} - I_{k} \end{bmatrix} \end{bmatrix}, \end{aligned}$$

where $\xi _1, \ldots , \xi _d \in \mathbb {T}\setminus \{1, -1\}$ are distinct eigenvalues of *U*, $I_{m}$ denotes the $m \times m$ identity matrix, and the block in the lower right corner might not appear or might appear as just $I_{\ell }$ or just $-I_{k}$, depending on whether 1 or $-1$ are eigenvalues of *U*. Of course $n_j$, $\ell$, and *k* represent the multiplicities of the respective eigenvalues and $2 n_1 + \cdots + 2 n_d + \ell + k = n$. One can now use Proposition 6.1 to prove the following.

#### Theorem 6.4

Suppose that *U* is an $n \times n$ unitary matrix with $U \cong U^{*}$ and *W* is a unitary matrix such that $W U W^{*} = U'$, where $U'$ is the matrix from (6.3). Then every $C \in {\mathscr {C}}_{c}(U)$ takes the form

$$\begin{aligned} C = W \begin{bmatrix} \begin{bmatrix} &{} V_{1} \\ V_{1}^{t} &{} \end{bmatrix} &{} &{} \\ &{} \ddots &{} &{} \\ &{} &{} &{} \begin{bmatrix} &{} V_{d} \\ V_{d}^{t} &{} \end{bmatrix}\\ &{} &{} &{} &{} \begin{bmatrix} Q_{\ell } &{} \\ &{} Q_k \end{bmatrix} \end{bmatrix} J W^{*}, \end{aligned}$$

where each $V_j$ is an $n_j \times n_j$ unitary matrix, $Q_{\ell }$, $Q_{k}$ are $\ell \times \ell$ and $k \times k$ (respectively) unitary matrices with $Q_{\ell }^{t} = Q_{\ell }$ and $Q_{k}^{t} = Q_{k}$ (in which only one or perhaps both might not appear depending whether 1 or $-1$ are eigenvalues of *U*), and *J* is the conjugation on $\mathbb {C}^n$ from (6.2).

### Unitary multiplication operators on $L^2(m, \mathbb {T})$

As discussed in [20, Example 5.16] there is model for any bilateral shift *U* on ${\mathcal {H}}$ as the multiplication operator $M_{\psi }$ on $L^2 = L^2(m, \mathbb {T})$, where $\psi$ is an inner function whose degree is that of the dimension of any wandering subspace for *U*. In this section, we give a concrete description of ${\mathscr {C}}_c(M_{\psi })$. If $J^\#$ is the conjugation on $L^2$ defined by $(J^\# f)(\xi ) =f^\#(\xi )= \overline{f(\bar{\xi })},$ and $C \in {\mathscr {C}}_c(M_{\psi })$, then $C J^\#$ is a unitary operator on $L^2$ for which $(C J^\#)M_{\psi } = M_{\psi } (C J^\#).$ This trick was used in several places [3, 4, 7]. The bounded operators on $L^2$ which commute with $M_{\psi }$, i.e., the *commutant* of $M_{\psi }$, were described in [19, Theorem 7.3].

Recall the known fact (see for example [20, Proposition 5.17]) that for an inner function $\psi$ we have the following orthogonal decomposition for $L^2$, namely,

6.5 $$\begin{aligned} L^2 = \bigoplus _{n = -\infty }^{\infty } \psi ^{n} {\mathcal {K}}_{\psi },\end{aligned}$$

where ${\mathcal {K}}_{\psi }:= H^2 \cap (\psi H^2)^{\perp }$ is the model space associated with $\psi$ (see [10] for a review of model spaces).

Let us set up some notation to be used below. For an inner function $\psi$, let $N:= {\text {dim}} {\mathcal {K}}_{\psi } \in \mathbb {N}\cup \{\infty \}$ and $\{h_j\}_{1 \leqslant j \leqslant N}$ be a fixed orthonormal basis for ${\mathcal {K}}_\psi$. Observe that *N* is finite if and only if $\psi$ is a finite Blaschke product with *N* zeros, repeated according to multiplicity [10, Prop. 5.19]. Also define

$$\begin{aligned} \bigoplus _{1 \leqslant j \leqslant N} L^2 = L^2 \oplus L^2 \oplus \cdots \oplus L^2. \end{aligned}$$

The norm of an ${\varvec{f}}= [f_{j}]_{1 \leqslant j \leqslant N}^{t}$ of $\bigoplus _{1 \leqslant j \leqslant N} L^2$ is $\Vert {\varvec{f}}\Vert := \Big (\sum _{1 \leqslant j \leqslant N} \Vert f_j\Vert ^{2}_{L^2}\Big )^{\frac{1}{2}}.$ When $N = \infty$, we need to assume that the sum defining $\Vert {\varvec{f}}\Vert$ above is finite. Furthermore, the operator $\bigoplus _{1 \leqslant j \leqslant N} M_{\xi }$ (called the *inflation* of the bilateral shift $M_{\xi }$ on $L^2$) is given by

$$\begin{aligned} \Big (\bigoplus _{1 \leqslant j \leqslant N} M_{\xi }\Big ) {\varvec{f}}(\xi ) = \xi {\varvec{f}}(\xi ) = [\xi f_{j}(\xi )]^{t}_{1 \leqslant j \leqslant N}. \end{aligned}$$

We also define

$$\begin{aligned} \ell ^{2}_{N}:= \Big \{\textbf{x} = [x_j]^{t}_{1 \leqslant j \leqslant N}, x_j \in \mathbb {C}: \Vert \textbf{x}\Vert _{\ell ^{2}_{N}} = \Big (\sum _{1 \leqslant j \leqslant N} |x_j|^2\Big )^{\frac{1}{2}} < \infty \Big \}. \end{aligned}$$

When $N = \infty$, this is the familiar sequence space $\ell ^2$. Finally, observe that

6.6 $$\begin{aligned} \bigoplus _{1 \leqslant j \leqslant N} M_{\xi } \cong {{\varvec{M}}}_{\xi }|_{{\mathscr {L}}^2(m, \ell ^{2}_{N})}. \end{aligned}$$

As a consequence, using the discussion from Sect. 4, note that

6.7 $$\begin{aligned} {\mathscr {C}}_{c}(M_{\psi }) \not = \varnothing . \end{aligned}$$

We will actually describe ${\mathscr {C}}_{c}(M_{\psi })$ below.

From [19] we have the unitary operator

6.8 $$\begin{aligned} W:L^2 \rightarrow \bigoplus _{1 \leqslant j \leqslant N}L^2, \quad Wf=[f_j]_{1 \leqslant j \leqslant N}^t, \end{aligned}$$

where $f=\sum _{1 \leqslant j \leqslant N}h_j \cdot (f_j \circ \psi )$ is a unique decomposition given by [19, Lemma 7.3]. Note that

6.9 $$\begin{aligned} f_j=\sum _{m=-\infty }^{\infty }a_{mj}\xi ^m \end{aligned}$$

and the coefficients $a_{mj}$ arise from the decomposition from (6.5) which yields the unique decomposition

6.10 $$\begin{aligned} f=\sum _{1 \leqslant j \leqslant N}h_j\sum _{m=-\infty }^{\infty }a_{mj}\psi ^m. \end{aligned}$$

Also recall from [19, Thm. 7.3] that

$$\begin{aligned} W^*[k_j]_{1 \leqslant j \leqslant N}^t=\sum _{1 \leqslant j \leqslant N}h_j \cdot (k_j\circ \psi ). \end{aligned}$$

Let *J* and $J^\#$ denote the standard conjugations on $L^2$ defined by $Jf(\xi )=\overline{f(\xi )}$ and $(J^\# f)(\xi )=f^\#(\xi ): = \overline{f(\bar{\xi })}.$ For our inner function $\psi$, observe that $\psi ^\# = J^\# \psi$ is also inner.

#### Proposition 6.11

For an inner function $\psi$ we have the following.

- $$J^{\#}{\mathcal {K}}_\psi = {\mathcal {K}}_{\psi ^{\#}}$$.
- If $\{h_j\}_{1 \leqslant j \leqslant N}$ is an orthonormal basis for ${\mathcal {K}}_\psi$ then $\{h_j^{\#}\}_{1 \leqslant j \leqslant N}$ is an orthonormal basis for ${\mathcal {K}}_{\psi ^{\#}}$.

#### Proof

Part (a) was shown in [3, Lemma 4.4] while part (b) is a consequence of the facts that conjugations preserve orthonormality (recall (2.1)). $\square$

Let $W_{\#}$ be the unitary operator from (6.8), where the inner function $\psi$ is replaced by $\psi ^{\#}$ and orthonormal basis and the orthonormal basis $\{h_j\}_{1 \leqslant j \leqslant N}$ is replaced by the orthonormal basis $\{h_j^{\#}\}_{1 \leqslant j \leqslant N}$, i.e.,

$$\begin{aligned} W_{\#}g=[g_j]_{1 \leqslant j \leqslant N}^t, \; \text{ where } \; g = \sum _{1 \leqslant j \leqslant N}h_j^{\#} \cdot (g_j\circ \psi ^{\#}). \end{aligned}$$

There are the two natural conjugations ${\varvec{J}}$ and ${\varvec{J}}^{\#}$ on $\bigoplus _{1 \leqslant j \leqslant N} L^{2}$ defined for each $\textbf{F} \in \bigoplus _{1 \leqslant j \leqslant N} L^{2}$, $\textbf{F}= [f_j]^{t}_{1 \leqslant j \leqslant N}$, by

$$\begin{aligned}{\varvec{J}}\textbf{F}&= [ {{\bar{f}}}_j]^{t}_{1 \leqslant j \leqslant N} =: \bar{{\textbf{F}}} \quad \text{ and } \quad {\varvec{J}}^\#\textbf{F} =[ f_j^\#]^{t}_{1 \leqslant j \leqslant N}=:{\textbf{F}}^\#. \end{aligned}$$

#### Proposition 6.12

Let $\psi$ be an inner function and $\{h_j\}_{1 \leqslant j \leqslant N}$ be an orthonormal basis for ${\mathcal {K}}_\psi$. Then we have the following.

- If $${\displaystyle f=\sum _{1 \leqslant j \leqslant N}h_j \cdot (f_j\circ \psi )}$$ then $${\displaystyle f^\#=J^{\#}f=\sum _{1 \leqslant j \leqslant N}h_j^{\#}\cdot (f_j^{\#}\circ \psi ^{\#})}$$.
- $$W_{\#}J^{\#}W^*={\varvec{J}}^{\#}$$.

#### Proof

Let $f\in L^2$ and observe from (6.9) and (6.10) that

$$\begin{aligned}J^{\#}f=\sum _{j=1}^{\infty }h_j^{\#}\sum \overline{a_{mj}}(\psi ^{\#})^m\quad \text {and}\quad f_j^{\#}=\sum _{m=-\infty }^{\infty }\overline{a_{mj}}\xi ^m.\end{aligned}$$

Hence $J^{\#}f=\sum _{j=1}^{\infty }h_j^{\#} \cdot (f_j^{\#}\circ \psi ^{\#}),$ which proves (a). The above also yields

$$\begin{aligned} W_{\#}J^{\#}W^*[f_j]_{1 \leqslant j \leqslant N}^t=W_{\#}J^{\#}f=[f_j^{\#}]_{1 \leqslant j \leqslant N}^t= {\varvec{J}}^{\#}[f_j]_{1 \leqslant j \leqslant N}^t, \end{aligned}$$

which proves (b). $$\square $$

#### Theorem 6.13

Suppose that $\psi$ is inner and $\{h_j\}_{1 \leqslant j \leqslant N}$ is an orthonormal basis for ${\mathcal {K}}_{\psi }$. Then we have the following,

- $${\mathscr {C}}_{c}(M_{\psi }) \not = \varnothing $$.
- $C \in {\mathscr {C}}_{c}(M_{\psi })$ if and only if there is a $\Phi = [\varphi _{i j}]_{1 \leqslant i, j \leqslant N} \in {\mathscr {L}}^{\infty }(m, \ell ^2_{N})$ such that
  6.14 $$\begin{aligned} \Phi (\xi )=\Phi (\bar{\xi }) \; \text{ and } \; \Phi ^{*}(\xi ) \Phi (\xi ) = I \; \text{ a.e. } \text{ on } \mathbb {T} \text{ and } \end{aligned}$$
  6.15 $$\begin{aligned} C f = \sum _{1 \leqslant j \leqslant N} ( f^\#_j \circ \psi ) \sum _{1 \leqslant k \leqslant N} h_k \cdot (\varphi _{k, j} \circ \psi )\end{aligned}$$
  for all $${\displaystyle f = \sum _{1 \leqslant j \leqslant N} h_j \cdot (f_j \circ \psi )\in L^2}$$.

#### Proof

Statement (a) follows from (6.7). To prove (b), observe that since *W* is a unitary operator, then ${{\widetilde{C}}}:=WCW^*$ is a conjugation on $\bigoplus _{1 \leqslant j \leqslant N} L^2$ (Lemma 2.3). If $C M_{\psi } = M_{{\psi }}C$, it follows from [19, Theorem 7.2(c)] that

$$\begin{aligned} {{\widetilde{C}}}\Big ( \bigoplus _{1 \leqslant j \leqslant N}M_{\xi }\Big ) = \Big (\bigoplus _{1 \leqslant j \leqslant N}M_{\xi }\Big ){{\widetilde{C}}}. \end{aligned}$$

Since the operator $\bigoplus _{1 \leqslant j \leqslant N}M_{\xi }$ on $\bigoplus _{j\geqslant 1}L^2$ is unitary equivalent to ${{\varvec{M}}}_{\xi }$ on ${\mathscr {L}}^2(m,\ell ^2_N)$ (recall (6.6)), the result [4, Theorem 4.3] says there is a $\Phi = [\varphi _{i j}]_{1 \leqslant i, j \leqslant N} \in {\mathscr {L}}^{\infty }(m, \ell ^2_{N})$ such that $\Phi (\xi )$ is unitary for a.e. $\xi \in \mathbb {T}$, ${{\varvec{M}}}_{\Phi }$ is ${\varvec{J}}^\#$–symmetric, and ${{\widetilde{C}}}={{\varvec{M}}}_\Phi {\varvec{J}}^\#.$ The unitary property gives $\Phi ^*(\xi )\Phi (\xi )=I$ and the ${\varvec{J}}^\#$–symmetry property gives $\Phi (\xi )=\Phi (\bar{\xi })$ a.e.  on $\mathbb {T}$. So far, we have shown that if *C* is a conjugation which commutes with $M_{\psi }$, then $W C W^{*} = {{\varvec{M}}}_{\Phi } {\varvec{J}}^{\#}$, where $\Phi$ satisfies the two properties from (6.14). Conversely suppose that $\Phi = [\varphi _{i j}]_{1 \leqslant i, j \leqslant N} \in {\mathscr {L}}^{\infty }(m, \ell ^2_{N})$ satisfies the two conditions from (6.14). The second condition will show that ${\varvec{J}}^{\#} {{\varvec{M}}}_{\Phi } {\varvec{J}}^{\#} = {{\varvec{M}}}_{\Phi }^{*}$ and combining this with the first condition will show that $\Phi$ is unitary valued a.e. The second property, along with Proposition 4.10 will show that ${{\varvec{M}}}_{\Phi } {\varvec{J}}^{\#}$ is a conjugation and belongs to ${\mathscr {C}}_{c}({{\varvec{M}}}_{\xi })$. By the discussion above, this says that $W^{*} ({{\varvec{M}}}_{\Phi } {\varvec{J}}^{\#}) W \in {\mathscr {C}}_{c}(M_{\psi })$.

Applying Proposition 6.12 we can verify the formula (6.15). Indeed, for each $f=\sum _{1 \leqslant j \leqslant N}h_j \cdot (f_j\circ \psi ) \in L^2$ we have

$$\begin{aligned} C f&= W^{*} {{{\varvec{M}}}}_{\Phi } {\varvec{J}}^{\#} W f\\&= W^{*} {{{\varvec{M}}}}_{\Phi } W_\# J^{\#} \Big (\sum _{1 \leqslant j \leqslant N}h_j \cdot (f_j\circ \psi )\Big )\\&= W^{*} {{{\varvec{M}}}}_{\Phi } W_\#\Big (\sum _{1 \leqslant j \leqslant N}h_j^{\#} \cdot (f_j^{\#}\circ \psi ^{\#})\Big )\\&= W^{*} [\varphi _{ij} ]_{1 \leqslant i, j \leqslant N}[ f^\#_j]_{1 \leqslant j \leqslant N}^{t}\\&= W^{*} \Big [\sum _{1 \leqslant i, j \leqslant N} \varphi _{1j} f^\#_j, \sum _{1 \leqslant i, j \leqslant N} \varphi _{2j} f^\#_j, \sum _{1 \leqslant i, j \leqslant N} \varphi _{3j} f^\#_j, \ldots \Big ]^{t}\\&= h_1\cdot \Big (\sum _{1 \leqslant i, j \leqslant N} \varphi _{1j} f^\#_j\Big )\circ \psi + h_2 \cdot \Big (\sum _{1 \leqslant i, j \leqslant N} \varphi _{2j} f^\#_j\Big ) \circ \psi + \ldots \\&= (f^\#_{1} \circ \psi ) \cdot \Big (\sum _{1 \leqslant i, j \leqslant N} h_{j} (\varphi _{j1} \circ \psi \Big ) + (f^\#_{2} \circ \psi ) \cdot \Big (\sum _{1 \leqslant i, j \leqslant N} h_{j} (\varphi _{j2} \circ \psi \Big ) + \cdots \end{aligned}$$

and this completes the proof. $\square$

#### Example 6.16

Consider the inner function $\psi (z) = z$. Here the associated unitary operator $M_{\psi }$ is merely *the* bilateral shift $M_{\xi }$ on $L^2$. In this case, ${\mathcal {K}}_{\psi } = \mathbb {C}$ (the constant functions). Moreover, $\psi ^{\#}(z) = z$ and the expansions from Proposition 6.12 are the standard Fourier expansions of an $f \in L^2$. Theorem 6.13 says that any $C \in {\mathscr {C}}_{c}(M_{\xi })$ takes the form $(C f)(\xi ) = u(\xi ) \overline{f(\bar{\xi })}$ for some $u \in L^{\infty }$ that is unimodular and satisfies $u(\xi ) = u(\bar{\xi })$ a.e.  on $\mathbb {T}$.

#### Example 6.17

Consider the inner function $\psi (z) = z^2$ as in [19, Example 7.7]. Then ${\mathcal {K}}_{\psi } = {\text {span}} \{1, z\} = \{h_1, h_2\}$. Furthermore, using the notation from this section,

$$\begin{aligned} f(\xi ) = h_{1}(\xi ) f_{1}(\xi ^2) + h_{2}(\xi ) f_{2}(\xi ^2) = f_{1}(\xi ^2) + \xi f_{2}(\xi ^2), \end{aligned}$$

where

$$\begin{aligned} f_1(\xi ) = \sum _{j = -\infty }^\infty \widehat{f}(2 j) \xi ^j \quad \text{ and } \quad f_{2}(\xi ) = \sum ^\infty _{j = -\infty } \widehat{f}(2 j + 1) \xi ^j. \end{aligned}$$

From here, one can check (Theorem 6.13) that every $C \in {\mathscr {C}}_s(M_{\xi ^2})$ takes the form

$$\begin{aligned} (C f)(\xi ) = { f_{1}^\#(\xi ^2)} (\varphi _{11}(\xi ^2) + \xi \varphi _{21}(\xi ^2)) + { f_{2}^\#(\xi ^2)} (\varphi _{12}(\xi ^2) + \xi \varphi _{22}(\xi ^2)), \end{aligned}$$

where $\varphi _{ij}$ are bounded measurable functions on $\mathbb {T}$ for which

6.18 $$\begin{aligned} \begin{bmatrix} \overline{ \varphi _{11}(\xi )} &{} \overline{ \varphi _{21}(\xi )}\\ \overline{ \varphi _{12}(\xi )} &{} \overline{ \varphi _{22}(\xi )} \end{bmatrix} \begin{bmatrix} \varphi _{11}(\xi ) &{} \varphi _{12}(\xi )\\ \varphi _{21}(\xi ) &{} \varphi _{22}(\xi ) \end{bmatrix}= \begin{bmatrix} 1 &{} 0\\ 0 &{} 1 \end{bmatrix}.\end{aligned}$$

and $\varphi _{ij}(\bar{\xi })=\varphi _{ij}(\xi )$, $i,j=1,2$, for a.e.  $\xi \in \mathbb {T}$. Condition (6.18) is equivalent to the conditions

$$\begin{aligned} |\varphi _{11}(\xi )|^2+|\varphi _{21}(\xi )|^2= 1, \\ |\varphi _{12}(\xi )|^2+|\varphi _{22}(\xi )|^2= 1, \\ \overline{ \varphi _{11}(\xi )}\varphi _{12}(\xi )+\overline{\varphi _{21}(\xi )}\varphi _{22}(\xi )=0. \end{aligned}$$

Fix the convention that $t={\text {Arg}}(\xi )\in (-\pi ,\pi ]$ and that $s(t),\alpha (t),\beta (t),\gamma (t),\delta (t)$ are any $2\pi$–periodic real-valued bounded measurable functions. Considering the moduli of the functions above, we obtain

$$\begin{aligned} 0\leqslant & {} s(t) \leqslant 1, \\ \varphi _{11}(\xi )= & {} e^{i\alpha (t)}s(t), \\ \varphi _{12}= & {} e^{i\beta (t)}\sqrt{1-s^2(t)}, \\ \varphi _{21}(\xi )= & {} e^{i\gamma (t)}\sqrt{1-s^2(t)}, \\ \varphi _{22}(\xi )= & {} e^{i\delta (t)}s(t). \end{aligned}$$

As to the arguments of the functions above, we obtain

$$\begin{aligned} \delta (t)=\beta (t))+\gamma (t)-\alpha (t)-\pi . \end{aligned}$$

Incorporating the conditions $\varphi _{ij}(\bar{\xi })=\varphi _{ij}(\xi )$, $i,j=1,2$, we obtain

$$\begin{aligned} \begin{bmatrix} \varphi _{11}(\xi ) &{} \varphi _{12}(\xi )\\ \varphi _{21}(\xi ) &{} \varphi _{22}(\xi ) \end{bmatrix}= \begin{bmatrix} e^{i\alpha (|t|)}s(|t|) &{} e^{i\beta (|t|)}\sqrt{1-s^2(|t|)}\\ e^{i\gamma (|t|)}\sqrt{1-s^2(|t|)} &{} -e^{i(\beta (|t|)+\gamma (|t|)-\alpha (|t|))}s(|t|) \end{bmatrix}.\end{aligned}$$

Finally, every conjugation $C \in {\mathscr {C}}_s(M_{\xi ^2})$ must take the form

$$\begin{aligned} (C f)(\xi ){} & {} = { f^\#_{1}(\xi ^2)}\Big ( e^{i\alpha (2|t|)}s(2|t|) + \xi e^{{i}(\gamma (2|t|))}\sqrt{1-s^2(2|t|)}\Big )\\ {}{} & {} \quad + { f^\#_{2}(\xi ^2)}\Big ( e^{{i}\beta (2|t|)}\sqrt{1-s^2(2|t|)} -\xi e^{i(\beta (2|t|)+\gamma (2|t|)-\alpha (2|t|))}s(2|t|)\Big ), \end{aligned}$$

where $t={\text {Arg}}(\xi )\in (-\pi ,\pi ]$ and $s(t),\alpha (t),\beta (t), \gamma (t)$ are any $2\pi$–periodic real bounded measurable functions.

#### Example 6.19

As a specific nontrivial example of a $C \in {\mathscr {C}}_{c}(M_{\xi ^2})$ we can take

$$\begin{aligned} (C f)(\xi ) = { f^\#_{1}(\xi ^2)} \big (\sin (2|t|) + \xi \cos (2t)\big ) + { f^\#_{2}(\xi ^2)}\big (\cos (2t) - \xi \sin (2|t|)\big ), \end{aligned}$$

where $t={\text {Arg}}(\xi )$ and $s(t)= \sin (t),\alpha (t)\equiv 0,\beta (t)\equiv 0,\gamma (t)\equiv 0$. For another nontrivial example of a $C \in {\mathscr {C}}_{c}(M_{\xi ^2})$, we set $s(t)\equiv s\in [0,1],\alpha (t)=\lambda t, \lambda \in \mathbb {R},\beta (t)\equiv 0,\gamma \equiv 0$ to get

$$\begin{aligned} (C f)(\xi ) = { f^\#_{1}(\xi ^2)} \big (s e^{i\lambda |t|}+ \xi \sqrt{1-s^2}\big ) + { f^\#_{2}(\xi ^2)}\big (\sqrt{1-s^2} - \xi e^{i\lambda |t|})\big ), \end{aligned}$$

where $$t={\text {Arg}}(\xi )$$.

### The bilateral shift on a $L^2(\mu , \mathbb {T})$ space

In Example 6.16${\mathscr {C}}_{c}(M_{\xi })$ for the bilateral shift $M_{\xi }$ on $L^2(m, \mathbb {T})$. This example contains a description of ${\mathscr {C}}_{c}(U)$ when $U = M_{\xi }$ on a more complicated $L^2(\mu , \mathbb {T})$ space. Let $g:[-1,1]\rightarrow [0, \infty )$ be defined piecewise by

$$\begin{aligned}g(t)=\left\{ \begin{array}{ll} \tfrac{3}{2}\,t^2, &{} {t\in [0,1],} \\ \frac{5}{2}\,t^4, &{} {t\in [-1,0].} \end{array} \right. \end{aligned}$$

If *dt* represents Lebesgue measure on $[-1,1]$, define the following measures on the Borel subsets $\Omega \subseteq [-1,1]$ by

$$\begin{aligned} {\widetilde{\mu }}_1(\Omega )=\int _\Omega g(t)\,dt, \quad {\widetilde{\mu }}_2(\Omega )=\int _\Omega g(-t)\,dt. \end{aligned}$$

One can verify that

$$\begin{aligned} {\widetilde{h}}(t)&:=\frac{d{\tilde{\mu }}_2}{d{\tilde{\mu }}_1}(t)=\frac{d{\tilde{\mu }}_2}{dt}\bigg (\frac{d{\tilde{\mu }}_1}{dt}\bigg )^{-1}(t)\\&= \left\{ \begin{array}{ll} \tfrac{5}{3} t^2, &{} {t\in [0,1],} \\ \tfrac{3}{5} t^{-2}, &{} {t\in [-1,0].}\\ \end{array} \right. \end{aligned}$$

Clearly ${\widetilde{h}}(t)\cdot {\widetilde{h}}(-t)=1$ on $[-1, 1]$. Now let

$$\begin{aligned} \gamma :[-1,1]\rightarrow {\mathbb {T}}, \quad \gamma (t)=\exp (2\pi it) \end{aligned}$$

and check that $\gamma ^{-1}(\xi )= \frac{{{\,\mathrm{\textrm{Arg}}\,}}\xi }{2\pi }$ for $\xi \in \mathbb {T}.$ Define measures $\mu _1,\mu _2 \in M_{+}(\mathbb {T})$ on Borel sets $\Omega \subseteq \mathbb {T}$ by

$$\begin{aligned} \mu _k(\Omega )=\widetilde{\mu }_k(\gamma ^{-1}(\Omega )), \quad k=1,2, \end{aligned}$$

and observe that $\mu _1^c=\mu _2$ and $\mu _2\ll \mu _1$. Moreover, we can write the Radon-Nikodym derivative

$$\begin{aligned} h(\xi ):=\frac{d\mu _2}{d\mu _1}(\xi )={\widetilde{h}}(\gamma ^{-1}(\xi ))=\big (\tfrac{5}{3}\big )^{{{\,\mathrm{\textrm{sgn}}\,}}({{\,\mathrm{\textrm{Arg}}\,}}\xi )}\, ({{\,\mathrm{\textrm{Arg}}\,}}\xi )^{2\,{{\,\mathrm{\textrm{sgn}}\,}}({{\,\mathrm{\textrm{Arg}}\,}}\xi ) }. \end{aligned}$$

From here one sees that $h(\xi )h(\bar{\xi })=1$ on $\mathbb {T}$ as seen in Proposition 3.3.

As in (4.9), define $J^{\#}$ on $L^2(\mu _1)$ by $(J^\# f)(\xi )= h( \xi )^{\frac{1}{2}}\ \overline{f(\overline{\xi })}.$ Then $J^\#$ is a conjugation and $J^\# M_\xi J^\#= M_\xi$. Moreover, Theorem 5.3 says that any conjugation *C* on $L^2(\mu _1)$ such that $C M_\xi C= M_\xi$ can be written as $C=uJ^\#$, where $u\in L^\infty (\mu _1)$ is unimodular and $u(\overline{\xi })=u(\xi )$ for $\mu _1$ a.e.  $\xi \in \mathbb {T}$.

### The Fourier transform

Let ${\mathcal {F}}$ denote the standard Fourier–Plancherel transform on $L^2(\mathbb {R})$. It is well known that ${\mathcal {F}}$ is unitary with spectrum $\{1, i, -1, -i\}.$ Moreover, the Hermite functions $\{H_n\}_{n \geqslant 0}$ form an orthonormal basis for $L^2(\mathbb {R})$ and ${\mathcal {F}} H_n = (-i)^n H_n$ for all $n \geqslant 0$, i.e., the Hermite functions form an eigenbasis for ${\mathcal {F}}$ [14, Ch.11]. A description of ${\mathscr {C}}_{s}({\mathcal {F}})$, the symmetric conjugations, was given in [19, Example 4.3]. In this example we work out ${\mathscr {C}}_{c}({\mathcal {F}})$, the commuting conjugations for ${\mathcal {F}}$. We first note that ${\mathcal {F}} \cong {\mathcal {F}}^{*}$ (Example 2.4). Thus, ${\mathscr {C}}_{c}({\mathcal {F}}) \not = \varnothing$ (Corollary 5.4).

To describe ${\mathscr {C}}_{c}({\mathcal {F}})$, we proceed as follows. Our discussion so far says that

6.20 $$\begin{aligned} L^2(\mathbb {R}) ={\mathscr {E}}_{1} \oplus {\mathscr {E}}_{-i} \oplus {\mathscr {E}}_{-1} \oplus {\mathscr {E}}_{i},\end{aligned}$$

where ${\mathscr {E}}_{\alpha } = \ker ({\mathcal {F}} - \alpha I)$. Define a conjugation *J* on $L^2(\mathbb {R})$ for which $J H_n = H_n$ for all $n \geqslant 0$ (initially define *J* on $H_n$ by $J H_n = H_n$ and extend antilinearly to all of $L^2(\mathbb {R})$).

If $\ell ^2 = \ell ^2(\mathbb {N}_0)$ is the classical sequence space with the standard orthonormal basis $\{\textbf{e}_n\}_{n \geqslant 0}$, and

$$\begin{aligned} V = V_{1} \oplus V_{-i} \oplus V_{-1} \oplus V_{i}, \end{aligned}$$

where $V_{1}$ is the unitary from ${\mathscr {E}}_{1}$ to $\ell ^2$ defined by $V_{1}(H_{4n}) = \textbf{e}_n$; $V_{-i}$ is the unitary from ${\mathscr {E}}_{-i}$ to $\ell ^2$ defined by $V_{-i}(H_{4 n + 1}) = \textbf{e}_n$; $V_{-1}$ is the unitary from ${\mathscr {E}}_{-1}$ to $\ell ^2$ defined by $V_{-1}(H_{4 n + 2}) = \textbf{e}_n$; $V_{i}$ is the unitary from ${\mathscr {E}}_{i}$ to $\ell ^2$ defined by $V_{i}(H_{4 n + 3}) = \textbf{e}_n$; then *V* is a unitary operator from $L^2(\mathbb {R})$ onto ${\mathscr {L}}^2(\mu , \ell ^2)$, where $\mu =\delta _{1}+\delta _{-i}+\delta _{-1}+\delta _{i}$.

Define a conjugation ${{\widetilde{J}}}$ on $\ell ^2$ by $\widetilde{J}(\textbf{e}_n)=\textbf{e}_n$ for all $n \geqslant 0$. Since $\mu ^c\ll \mu$ we can define a conjugation ${\widetilde{{\varvec{J}}}}^\#$ on ${\mathscr {L}}^2(\mu , \ell ^2)$ such that $(V{\mathcal {F}}V^{*}){\widetilde{{\varvec{J}}}}^\#= {\widetilde{{\varvec{J}}}}^\# (V{\mathcal {F}}V^{*})$ by (4.6). In other words, with respect to the orthogonal decomposition

$$\begin{aligned} {\mathscr {L}}^2(\mu , \ell ^2) = {\mathscr {L}}^2(\delta _1, \ell ^2) \bigoplus {\mathscr {L}}^2(\delta _{-i}, \ell ^2) \bigoplus {\mathscr {L}}^2(\delta _{-1}, \ell ^2) \bigoplus {\mathscr {L}}^2(\delta _i, \ell ^2), \end{aligned}$$

the conjugation ${\widetilde{{\varvec{J}}}}^{\#}$ can be written in matrix form as

$$\begin{aligned} {\widetilde{{\varvec{J}}}}^\#= \begin{bmatrix} {{\widetilde{J}}} &{}\quad 0 &{}\quad 0 &{}\quad 0\\ 0 &{}\quad 0 &{}\quad 0 &{}\quad {{\widetilde{J}}} \\ 0 &{}\quad 0 &{}\quad {{\widetilde{J}}} &{}\quad 0 \\ 0 &{}\quad {{\widetilde{J}}} &{}\quad 0 &{}\quad 0 \end{bmatrix}. \end{aligned}$$

The conjugation $V^{*}{\varvec{J}}^\#V$ commutes with ${\mathcal {F}}$ and it can be written with respect to the Hermite basis as

$$\begin{aligned}{\varvec{J}}^\#H_{4n+k}:= W^{*}{\widetilde{{\varvec{J}}}}^\#W H_{4n+k}=\left\{ \begin{array}{ll} H_{4n+k}, &{}\quad {k=0,} \\ H_{4n+k+2}, &{}\quad {k=1,} \\ H_{4n+k}, &{}\quad {k=2,} \\ H_{4n+k-2}, &{}\quad k=3. \end{array} \right. \end{aligned}$$

Therefore, the matrix representation of ${\varvec{J}}^{\#}$ with respect to the orthogonal decomposition in (6.20) is

$$\begin{aligned} {\varvec{J}}^\#= \begin{bmatrix} J &{}\quad 0 &{}\quad 0 &{}\quad 0\\ 0 &{}\quad 0 &{}\quad 0 &{}\quad J \\ 0 &{}\quad 0 &{}\quad J &{}\quad 0 \\ 0 &{}\quad J &{}\quad 0 &{}\quad 0 \end{bmatrix}. \end{aligned}$$

Moreover, by Theorem 5.3, any conjugation ${{\widetilde{C}}}$ on ${\mathscr {L}}^2(\mu , \ell ^2)$ such that

$$\begin{aligned} {{\widetilde{C}}}(V{\mathcal {F}}V^{*})=(V{\mathcal {F}}V^{*}){{\widetilde{C}}} \end{aligned}$$

can be represented by the matrix

$$\begin{aligned} {{\widetilde{C}}}= \begin{bmatrix} {{\widetilde{U}}}_{1}{{\widetilde{J}}} &{}\quad 0 &{}\quad 0 &{}\quad 0\\ 0 &{}\quad 0 &{}\quad 0 &{}\quad {{\widetilde{U}}}_{-i}{{\widetilde{J}}} \\ 0 &{}\quad 0 &{}\quad {{\widetilde{U}}}_{-1}{{\widetilde{J}}} &{}\quad 0 \\ 0 &{}\quad {{\widetilde{U}}}_i{{\widetilde{J}}} &{}\quad 0 &{}\quad 0 \\ \end{bmatrix}, \end{aligned}$$

where ${{\widetilde{U}}}_1,{{\widetilde{U}}}_{-i},\widetilde{U}_{-1},{{\widetilde{U}}}_{i}$, are unitary operators on $\ell ^2$ and

$$\begin{aligned} {{\widetilde{J}}}{{\widetilde{U}}}_1{{\widetilde{J}}}={{\widetilde{U}}}_{1}^{*}, \; {{\widetilde{J}}}{{\widetilde{U}}}_{-1}{{\widetilde{J}}}={{\widetilde{U}}}_{-1}^{*}, \; {{\widetilde{J}}}{{\widetilde{U}}}_{i}{{\widetilde{J}}} ={{\widetilde{U}}}_{-i}^{*}. \end{aligned}$$

The first two identities say that the unitary operators ${{\widetilde{U}}}_1$ and ${{\widetilde{U}}}_{-1}$ are represented by with respect to the basis $\{\textbf{e}_n\}_{n \geqslant 0}$ by a matrix with real entries. The last identity says that the matrix representations in the basis $\{\textbf{e}_n\}_{n \geqslant 0}$ of ${{\widetilde{U}}}_{i}$ and ${{\widetilde{U}}}_{-i}$ satisfy $\langle {{\widetilde{U}}}_{-i}\textbf{e}_m,\textbf{e}_n\rangle = \overline{\langle {\widetilde{U}}_{i}^{*} \textbf{e}_{m}, \textbf{e}_{n}\rangle ,}$ which we write as $\widetilde{U}_{-i}={{\widetilde{U}}}_{i}^{\#}$. Therefore, any conjugation ${{\widetilde{C}}}$ on ${\mathscr {L}}^2(\mu , \ell ^2)$ such that ${{\widetilde{C}}}(V{\mathcal {F}}V^{*})=(V{\mathcal {F}}V^{*}){{\widetilde{C}}}$ can be represented as

$$\begin{aligned} {{\widetilde{C}}}= \begin{bmatrix} {{\widetilde{U}}}^\mathbb {R}_{1}{{\widetilde{J}}} &{}\quad 0 &{}\quad 0 &{}\quad 0\\ 0 &{}\quad 0 &{}\quad 0 &{}\quad {{\widetilde{U}}}_{i}^{\#}{{\widetilde{J}}} \\ 0 &{}\quad 0 &{}\quad {{\widetilde{U}}}^\mathbb {R}_{-1}{{\widetilde{J}}} &{}\quad 0\\ 0 &{}\quad {{\widetilde{U}}}_i{{\widetilde{J}}} &{}\quad 0 &{}\quad 0 \\ \end{bmatrix}, \end{aligned}$$

where $U^\mathbb {R}_1$ and $U^\mathbb {R}_{-1}$ are arbitrary unitary operators on $\ell ^2$ whose matrix representations with respect to $\{\textbf{e}_n\}_{n \geqslant 0}$ have real entries and ${{\widetilde{U}}}_i$ is arbitrary. Finally, a conjugation *C* on $L^2(\mathbb {R})$ fulfils the condition $C{\mathcal {F}}={\mathcal {F}} C$ if and only if it is represented with respect to the decomposition in (6.20) as

$$\begin{aligned} C= \left[ \begin{array}{cccc} U^\mathbb {R}_{1} J &{}\quad 0 &{}\quad 0 &{}\quad 0\\ 0 &{}\quad 0 &{}\quad 0 &{}\quad U_{i}^{\#} J \\ 0 &{}\quad 0 &{}\quad U^\mathbb {R}_{-1} J &{}\quad 0 \\ 0 &{}\quad U_i J &{}\quad 0 &{}\quad 0 \\ \end{array} \right] = \left[ \begin{array}{cccc} U^\mathbb {R}_{1} &{}\quad 0 &{}\quad 0 &{}\quad 0\\ 0 &{}\quad 0 &{}\quad 0 &{}\quad U_{i}^{\#} \\ 0 &{}\quad 0 &{}\quad U^\mathbb {R}_{-1} &{}\quad 0\\ 0 &{}\quad U_i &{}\quad 0 &{}\quad 0 \\ \end{array} \right] \, J, \end{aligned}$$

where $U^\mathbb {R}_1, U^\mathbb {R}_{-1}$ are arbitrary unitary operators on their respective eigenspaces $\ker ({\mathcal {F}} - I)$ and $\ker ({\mathcal {F}} + I)$ which are represented in terms of the basis $\{H_{4n}\}_{n \geqslant 0}$ and $\{H_{4n+2}\}_{n \geqslant 0}$ by real matrices, $U_i$ is an arbitrary unitary operator on $\ker ({\mathcal {F}} + iI)$ and $U_{i}^{\#}$ is the unitary operator on $\ker ({\mathcal {F}} - i I)$ defined by $\langle U_{i}^{\#}H_{4\,m+3},H_{4n+3}\rangle =\overline{\langle U_{i}^{*}H_{4\,m+1}, H_{4 n+1}\rangle }, \quad m, n \geqslant 0.$

### The Hilbert transform

Suppose ${\mathscr {H}}$ is the Hilbert transform on $L^2(\mathbb {R})$. Since the spectrum of ${\mathscr {H}}$ is $\{i, -i\}$ then $L^2(\mathbb {R}) = {\mathscr {E}}_{i} \bigoplus {\mathscr {E}}_{-i}$ and ${\mathscr {E}}_{i}$ has orthonormal basis ${\mathscr {B}}_{i} = \{f_n\}_{n \geqslant 1}$

$$\begin{aligned} f_n(x) = \frac{1}{\sqrt{\pi }} \frac{(x + i)^{n - 1}}{(x - i)^{n}} \end{aligned}$$

and ${\mathscr {E}}_{-i}$ has orthonormal basis ${\mathscr {B}}_{-i} = \{g_n\}_{n \geqslant 1}$

$$\begin{aligned} g_{n}(x) = \frac{1}{\sqrt{\pi }} \frac{(x - i)^{n-1}}{(x + i)^{n}}. \end{aligned}$$

See [14, Ch. 12] for details. In this example we will describe ${\mathscr {C}}_c({\mathscr {H}})$. Note first that ${\mathscr {C}}_c({\mathscr {H}})\not =\varnothing$ (recall Example 2.4 and thus ${\mathscr {H}}\cong {\mathscr {H}}^*$).

Similarly as was done with the Fourier transform, we can identify $L^2(\mathbb {R})$ with ${\mathscr {L}}^2(\mu , \ell ^2)$, where $\mu =\delta _i+\delta _{-i}$. Then, the conjugation ${\varvec{J}}^\#$ given by equality (4.6) is an antilinear extension of operator ${\varvec{J}}^\#f_n=g_n,\ {\varvec{J}}^\#g_n=f_n,\ n\geqslant 1$. Putting this in matrix form

$$\begin{aligned} {\varvec{J}}^\# = \begin{bmatrix} 0&{}\quad J \\ J&{}\quad 0 \end{bmatrix}, \end{aligned}$$

where *J* is a conjugation on $L^2(\mathbb {R})$ which fixes all elements of ${\mathscr {B}}_{i}$ and ${\mathscr {B}}_{-i}$.

Moreover, by Theorem 5.3, any conjugation *C* on $L^2(\mathbb {R})$ with $C {\mathscr {H}} C = {\mathscr {H}}^{*}$ must take the (block) form

$$\begin{aligned} C = \begin{bmatrix} U_i &{}\quad 0\\ 0&{}\quad U_{i}^\# \end{bmatrix} \begin{bmatrix} 0 &{}\quad J\\ J&{}\quad 0 \end{bmatrix}=\begin{bmatrix} 0 &{}\quad U_iJ\\ U_i^\#J&{}\quad 0 \end{bmatrix}, \end{aligned}$$

where $U_i$ is arbitrary unitary operator on ${\mathscr {E}}_i$ and $U_i^\#\in {\mathcal {B}}({\mathscr {E}}_{-i})$ defined as $\langle U_i^\#g_m,g_n\rangle =\overline{\langle U_i^*f_m,f_n\rangle }$ similar to the previous example.

## A remark about invariant subspaces

The first paper in this series [19] classified, for a fixed unitary operator *U* on ${\mathcal {H}}$, the subspaces ${\mathcal {M}}$ of ${\mathcal {H}}$ for which $C {\mathcal {M}} \subseteq {\mathcal {M}}$ for every ${\mathscr {C}}_{s}(U)$ (the symmetric conjugations for *U*). These turned out to be the hyperinvariant subspaces for *U*. What are the subspaces ${\mathcal {M}}$ for which $C {\mathcal {M}} \subseteq {\mathcal {M}}$ for every $C \in {\mathscr {C}}_{c}(U)$ (the commuting conjugations for *U*)? We have seen some partial results in this paper (see for example Proposition 3.7 and Corollary 3.9). We present some positive result in this direction. However, we do not have so pleasant characterization as in symmetric case. Generally these subspaces seem complicated to be simply described in the abstract situation. The difficulties, which can be came across even for multiplication operator, will be seen in Example 7.4. Of course, we need to have the natural assumption that ${\mathscr {C}}_{s}(U)\not =\varnothing$. We start a characterization in the special case where $U = {{\varvec{M}}}_{\xi }$ on ${\mathscr {L}}^{2}(\mu , {\mathcal {H}})$. Recall the notation from Proposition 4.5. Then we have to assume that $\mu ^c\ll \mu$.

### Theorem 7.1

Suppose $\mu \in M_{+}(\mathbb {T})$ such that $\mu ^{c} \ll \mu$ and ${\mathcal {H}}$ is a Hilbert space. For a subspace ${\mathcal {K}}$ of ${\mathscr {L}}^2(\mu , {\mathcal {H}})$ the following are equivalent.

- $${\mathfrak {C}} {\mathcal {K}} \subseteq {\mathcal {K}}$$ for every $${\mathfrak {C}} \in {\mathscr {C}}_{c}({{\varvec{M}}}_{\xi })$$;
- For any fixed conjugation *J* on ${\mathcal {H}}$ and ${\varvec{J}}^{\#}$ defined as in (4.6), subspace ${\mathcal {K}}$ is invariant for ${\varvec{J}}^{\#}$ and every ${{\varvec{M}}}_{{\textbf{F}}}$, where ${\textbf{F}}$ belongs to
  $$\begin{aligned} {\mathscr {L}}^{\infty }_{c}(\mu , {\mathcal {B}}({\mathcal {H}})):= \{ {\textbf{F}} \in {\mathscr {L}}^{\infty }(\mu , {\mathcal {B}}({\mathcal {H}})): J {\textbf{F}}(\xi ) J = {\textbf{F}}(\xi )^{\#} \text{ for } \mu \text{-a.e. } \xi \in \mathbb {T}\}. \end{aligned}$$

The proof of Theorem 7.1 requires a decomposition theorem from [22, pf.  of Corollary 3.19]. We include a proof for completeness and since the particular form of the decomposition is important for the proof of Theorem 7.1.

### Lemma 7.2

Any $A \in {\mathcal {B}}({\mathcal {H}})$ can be expressed as a positive constant times the sum of four unitary operators on ${\mathcal {H}}$.

### Proof

Define

$$\begin{aligned} H=\frac{1}{2 \Vert A\Vert }(A+A^*) \; \; \text{ and } \; \; K= \frac{1}{2 i \Vert A\Vert }(A-A^*) \end{aligned}$$

and notice that *H* and *K* are selfadjoint contractions and thus $I - H$ and $I - K$ are positive and hence have unique positive square roots. Thus,

$$\begin{aligned} U_{1,2}=H\pm i(I-H^2)^{\frac{1}{2}} \; \; \text{ and } \; \; U_{3,4}=i K\pm (I-K^2)^{\frac{1}{2}} \end{aligned}$$

are four unitary operators which satisfy

$$\begin{aligned} A&=\frac{\Vert A\Vert }{2}(U_1+U_2+U_3+U_4). \\ \end{aligned}$$

$$\square $$

### Proof of Theorem 7.1

The proof of (b) $\Longrightarrow$ (a) follows from a special case of Theorem 5.3. For the proof of (a) $\Longrightarrow$ (b), we begin with the fact that since ${\varvec{J}}^{\#} \in {\mathscr {C}}_{c}({{\varvec{M}}}_{\xi })$ then ${\mathcal {K}}$ is invariant for ${\varvec{J}}^{\#}$. Moreover, by Theorem 5.3 any ${\mathfrak {C}} \in {\mathscr {C}}_{c}({{\varvec{M}}}_{\xi })$ can be written as ${\mathfrak {C}} = {{\varvec{M}}}_{{{\varvec{U}}}} {\varvec{J}}^{\#}$ for some ${{\varvec{U}}}\in {\mathscr {L}}^{\infty }(\mu , {\mathcal {B}}({\mathcal {H}}))$ that is unitary valued $\mu$-a.e.  and satisfies $J {{\varvec{U}}}(\xi ) J = {{\varvec{U}}}(\xi )^{\#}$ for $\mu$-a.e.  $\xi$. Thus, ${\mathcal {K}}$ is invariant for ${{\varvec{M}}}_{{{\varvec{U}}}} {\varvec{J}}^{\#}$ and thus ${{\varvec{M}}}_{{{\varvec{U}}}}$. Apply Lemma  7.2 to any ${\textbf{F}} \in {\mathscr {L}}^{\infty }_{c}(\mu , {\mathcal {B}}({\mathcal {H}}))$ to see that ${\mathcal {K}}$ is invariant for every ${{\varvec{M}}}_{{\textbf{F}}}$ where ${\textbf{F}} \in {\mathscr {L}}^{\infty }_{c}(\mu , {\mathcal {B}}({\mathcal {H}}))$. $\square$

The example bellow shows that more pleasant characterization of subspaces which are invariant for all commuting conjugation, even in scalar case of multiplication operators, will be difficult to find.

### Remark 7.3

In the scalar case ${\mathcal {H}}= \mathbb {C}$, note that ${\mathscr {L}}^{\infty }(\mu , {\mathcal {B}}(\mathbb {C})) = \{v \in L^{\infty }(\mu ): v(\xi ) = v(\overline{\xi }) \hbox {for} \mu \hbox {-a.e.} \xi \in \mathbb {T}\}.$

### Example 7.4

For *the* bilateral shift $M_{\xi }$ on $L^2 = L^2(m, \mathbb {T})$, we know that every $C \in {\mathscr {C}}_{c}(M_{\xi })$ takes the form $M_{u} J$, where $(J f)(\xi ) = f(\xi )$ and $u \in L^{\infty }$ such that $u(\xi )$ is unimodular and $u(\xi ) = u(\overline{\xi })$ a.e. One can check that examples of subspaces that are invariant for every $C \in {\mathscr {C}}_{c}(M_{\xi })$ include

- $$\{g \in L^2: g(e^{i t}) = 0, |t| \geqslant \tfrac{\pi }{2}, g(e^{i t}) = g(e^{-it}), |t| < \tfrac{\pi }{2}\};$$
- $$\{g \in L^2: g(e^{i t}) = 0, |t| \geqslant \tfrac{\pi }{2}, g(e^{i t}) = -g(e^{-it}), |t| < \tfrac{\pi }{2}\};$$
- $$\{g \in L^2: g(e^{i t}) = 0, |t| \geqslant \tfrac{\pi }{2}, g(e^{i t}) = g(e^{-it}), \tfrac{\pi }{4}< |t|< \tfrac{\pi }{2}, g(e^{it}) = -g(e^{-it}), |t| < \tfrac{\pi }{4}\}.$$

The variety of these spaces convinces us that a concise description of the *C*-invariant subspaces for every $C \in {\mathscr {C}}_{c}(M_{\xi })$ seems difficult.

We finish with a characterization using the spectral multiplicities and Theorems 5.3 and 7.1

### Theorem 7.5

Suppose *U* is a unitary operator on ${\mathcal {H}}$ with ${\mathscr {C}}_{c}(U)\not =\varnothing$ and let ${\mathcal {K}}$ be a closed subspace of ${\mathcal {H}}$. With the notation as in (1.3), the following are equivalent.

- $${C} {\mathcal {K}} \subseteq {\mathcal {K}}$$ for every $${C} \in {\mathscr {C}}_{c}(U)$$;
- ${\mathcal {I}}{\mathcal {K}}=\bigoplus {\mathcal {K}}_i$, ${\mathcal {K}}_i\subseteq {\mathcal {H}}_i$, where ${{\varvec{C}}}^i {\mathcal {K}}_i\subseteq {\mathcal {K}}_i$, for all *i* and all ${{\varvec{C}}}^i\in {\mathscr {C}}_{c}({{\varvec{M}}}_{\xi }^{(i)})$;
- ${\mathcal {I}}{\mathcal {K}}=\bigoplus {\mathcal {K}}_i$, ${\mathcal {K}}_i\subseteq {\mathcal {H}}_i$, and all *i* and for any fixed conjugation $J_i$ on ${\mathcal {H}}_i$ and ${{\varvec{J}}^{\#}}^{(i)}$ defined as in (4.6), each subspace ${\mathcal {K}}_i$ is invariant for ${{\varvec{J}}^{\#}}^{(i)}$ and every ${{\varvec{M}}}_{{\textbf{F}}_i}$, where ${\textbf{F}}_i$ belongs to
  $$\begin{aligned} {\mathscr {L}}^{\infty }_{c}(\mu _i, {\mathcal {B}}({\mathcal {H}}_i)):= \{ {\textbf{F}}_i \in {\mathscr {L}}^{\infty }(\mu _i, {\mathcal {B}}({\mathcal {H}}_i)): J_i {\textbf{F}}_i(\cdot ) J_i = {\textbf{F}}_i(\cdot )^{\#},\ \mu _i\, a.e.\}. \end{aligned}$$

### Proof

Lemma 5.2 says that $\mu _i^c\ll \mu _i$ for each *i*. Note also that ${\mathcal {K}}$ is invariant for $C\in {\mathscr {C}}_{c}(U)$ if and only if ${\mathcal {I}}{\mathcal {K}}$ is invariant for ${\mathcal {I}}C{\mathcal {I}}^*\in {\mathscr {C}}_{c}(\widetilde{{{\varvec{M}}}}_\xi )$. Moreover, by Theorem 5.3, each $C\in {\mathscr {C}}_{c}(U)$ can be expressed as $C={\mathcal {I}}^* \big (\bigoplus {{\varvec{C}}}^i\big ){\mathcal {I}}$, where ${{\varvec{C}}}^i\in {\mathscr {C}}_{c}({{\varvec{M}}}_\xi ^{(i)})$. Observe that if $\bigoplus {{\varvec{C}}}^i\in {\mathscr {C}}_c(\widetilde{ {{\varvec{M}}}}_\xi )$, then $\bigoplus \epsilon _i{{\varvec{C}}}^i\in {\mathscr {C}}_c(\widetilde{ {{\varvec{M}}}}_\xi )$ for any choice $\epsilon _i=\pm 1$ and also recall that if subspace is invariant for a conjugation its orthogonal complement is also invariant. Therefore, ${\mathcal {I}}{\mathcal {K}}=\bigoplus {\mathcal {K}}_i$ where ${{\varvec{C}}}^i {\mathcal {K}}_i\subseteq {\mathcal {K}}_i$ for all ${{\varvec{C}}}^i\in {\mathscr {C}}_{c}({{\varvec{M}}}_{\xi }^{(i)})$. This proves the equivalence of (a) and (b). The equivalence of (b) and (c) follows from Theorem 7.1. $\square$

## Data Availability

No datasets were generated or analysed during the current study.
